# A burden of nerve injury from a global perspective, 1990–2021: an analysis of incidence, prevalence, and years lived with disability

**DOI:** 10.3389/fneur.2025.1669662

**Published:** 2026-01-06

**Authors:** Shunan Shi, Jiahao Tang, Yuexin Lu, Shanhu Xu, Ming Wang, Shu Wan

**Affiliations:** Brain Center, Affiliated Zhejiang Hospital, Zhejiang University School of Medicine, Hangzhou, Zhejiang, China

**Keywords:** nerve injury, global health, burden of disease, epidemiology, prediction

## Abstract

**Objective:**

To provide a solid foundation for the prevention, treatment, and reduction of the incidence of nerve injury, we explain and update the changes in the epidemiology of nerve injury from 1990 to 2021.

**Methods:**

We did our analysis employing the 2021 Global Burden of Disease, Injury and Risk Factor Study to calculate the incidence, prevalence and YLDs of nerve injury between 1990 and 2021 at the global, regional and national levels. The analysis was done according to gender, age group and region in layers.

**Results:**

In 2021, globally, there were 4.13 million [95% uncertainty interval (UI): 3.11–5.56] instances of nerve injury, featuring an age-standardized incidence and prevalence of 53 cases per 100,000 people, respectively (95% UI: 40–72) and 51 cases per 100,000 people (95% UI: 47–57). The global age-standardized incidence had an Average Annual Percent Change (AAPC) of −1.03% (95% UI: −1.38 − 0.68, *p* < 0.01), and the prevalence had an AAPC of −0.99% (95% UI, −1.06 − 0.93, *p* < 0.01), both showing a slightly decreasing trend. In 2021, nerve injury resulted in 440,000 (95% UI, 0.3–0.6) YLDs, with an age-standardized rate of 5.26 cases per 100,000 people (95% UI, 3.63–7.2). In 2021, the countries with relatively higher rates are predominantly located in the Caribbean and Oceania. The main causes of nerve injury are falls and exposure to mechanical forces. The global age-standardized incidence in 2021 is higher in men than in women. In most regions, changes in aging, epidemiological situations, and population characteristics are the main factors contributing to the variations in the incidence rate of nerve injury.

**Conclusion:**

This study focused on the occurrence of nerve injury from 1990 to 2021, and discovered that the global incidence rate went down a little, different SDI areas and regions had big differences, and the prevalence rate increased in some regions. It provides strong support for further exploring the causes of nerve injury and formulating future policies and protection measures.

## 1 Introduction

In recent years, nerve injury has emerged as an increasingly prominent global public health concern, posing a substantial threat to human health and well-being. Mounting evidence indicates that nerve injury not only undermines patients’ quality of life but also frequently induces long-term impairments in physical function. This imposes enormous economic pressure and heavy caregiving burdens on both societies and families (1). According to incomplete statistics, the direct and indirect economic losses attributable to nerve injury exceed hundreds of billions of US dollars annually, underscoring its profound implications for the stable operation of the global economy and communities (2).

For decades, the prevention and management of nerve injury have remained a pivotal focus in the medical field. The formulation of effective prevention and control strategies necessitates a comprehensive understanding of its epidemiological characteristics, etiological factors, and high-risk populations. Regrettably, significant limitations persist in the current epidemiological data on nerve injury. Inconsistent data standards have hindered cross-country and cross-regional comparative analyses, severely impeding the development of globally collaborative response strategies (3–5).

Recent epidemiological investigations have revealed the complex multifactorial nature of nerve injury predisposition. The global aging demographic shift, accompanied by age-related physiological decline, lifestyle-driven changes in disease patterns, and the dynamic impacts of population structure transitions, have emerged as key contributors to the rising incidence of nerve injury worldwide (6). Notably, the mechanisms by which these factors exert their effects are not uniform but exhibit substantial heterogeneity across geographical regions, socioeconomic gradients, genders, and age groups.

While previous iterations of the Global Burden of Disease (GBD) study have delineated certain epidemiological features of nerve injury, such as incidence and prevalence trends between 1990 and 2021, considerable knowledge gaps remain. In particular, research on age-specific pathogenic mechanisms of nerve injury remains insufficient, precluding the accurate characterization of nerve vulnerability trajectories from childhood through old age. To address these limitations, the present study leverages the latest GBD 2021 dataset to conduct a comprehensive multilevel analysis of nerve injury at the global, regional, and national levels. This study systematically describes the spatiotemporal evolution of nerve injury incidence, prevalence, and years lived with disability (YLDs) from 1990 to 2021, and provides an in-depth analysis of gender- and age-specific differences in injury mechanisms across regions with varying Socio-demographic Index (SDI) levels. The overarching objective is to furnish robust evidence for the precise prevention, control, and clinical management of nerve injury globally, thereby facilitating the optimization of health resource allocation and enhancing international collaboration in addressing this pressing global health challenge.

## 2 Method

### 2.1 Overview

This study adheres to the Guidelines for Accurate and Transparent Health Estimates Reporting (GATHER) and the Strengthening the Reporting of Observational Studies in Epidemiology (STROBE) statement to conduct a comprehensive analysis of the incidence, prevalence, and YLDs of nerve injury (7, 8). Within the research framework of the GBD 2021 study (9), nerve injury is not classified as a direct cause of death; therefore, the assessment of nerve injury burden in this study focuses primarily on incidence, prevalence, and YLDs, and does not include mortality or years of life lost (YLLs) at the current stage.

In the GBD study framework, the core calculation formula for YLDs is defined as: YLDs = Prevalence of a specific disease or injury × Corresponding disability weight (DW). Notably, the independent assignment of DWs for distinct health outcomes serves as the pivotal mechanism to ensure both the independence and additivity of YLD estimates across different diseases and injuries (9, 10).

### 2.2 Case definitions

Drawing on the methodological framework of the GBD 2021 study (7), the definition of nerve injury in this investigation is based on the 9th Edition of the International Classification of Diseases (ICD-9) and the 10th Edition of the International Classification of Diseases (ICD-10). This classification system was previously elaborated in the GBD 2016 study published in The Lancet Neurology (11). The specific ICD-9 codes adopted cover the range of 950.0–953.9, including codes for injuries to the optic nerve and pathways (950.0–950.9), injuries to cranial nerves (951.0–951.9), spinal cord injuries without evidence of spinal bone injury (952.0–952.9), and injuries to nerve roots and spinal plexus (953.0–953.9) (12). Corresponding ICD-10 codes mainly include the S04, S74, and S84 code ranges, such as codes for injuries to the optic tract and pathways (S04.039A), facial nerve (S04.51XS) in the head region, injuries to nerves at the hip and thigh level (S74.8X9A, S74.90XA), and injuries to the tibial nerve, peroneal nerve at the lower leg level (S84.00XA, S84.10XA) (13).

Furthermore, the GBD 2021 study clarifies that nerve impairment is categorized as a characteristic of injury rather than a causative factor of injury (7). It is important to emphasize that this investigation only includes cases of traumatic nerve injury (e.g., those caused by falls and accidents) and excludes non-traumatic cases (e.g., diabetic neuropathy). This exclusion criterion is based on the fact that non-traumatic neuropathies arise from metabolic disorders or other internal pathogenic factors, which do not align with the classification of nerve injury as a “nature-of-injury” associated with external traumatic causes in the GBD 2021 framework. For example, when a fall results in nerve injury, the fall is defined as the cause of injury, while the nerve injury itself is regarded as the nature of the injury.

### 2.3 Calculation of incidence, prevalence and YLD

The data processing procedures for calculating the incidence, prevalence, and YLDs of nerve injury in this study are consistent with the methodological structure of the GBD 2021 study (7). The incidence, prevalence, and YLDs related to nerve injury were estimated using DisMod-MR, a Bayesian meta-regression modeling tool (14). Uncertainty intervals (UIs) were generally derived from 1,000 draw-level estimations for each parameter. Specifically, the 95% UI was defined by the 25th and 975th values among the 1,000 ordered estimations.

### 2.4 Statistical analysis

First, based on the GBD 2021 location classification framework (7), we calculated the absolute values and age-standardized rates (ASRs) of incidence, prevalence, and YLDs of nerve injury across 5 SDI regions and 21 GBD regions in 2021. The age-standardized rate was calculated using the formula: ASR = *Σ* (Age-specific rate × Age weight), where the age weights were derived from the GBD 2021 World Population Standard (15).

Second, following the GBD 2021 analytical framework (7), we used the percentage change in age-standardized rates of incidence, prevalence, and YLDs to quantify trends in nerve injury burden from 1990 to 2021. A percentage change with a 95% UI that did not cross zero was considered statistically significant; otherwise, the change was deemed non-significant (16).

Third, based on the GBD 2021 cause hierarchy, our analysis identified the primary causes of the age-standardized incidence of nerve injury worldwide in 2021, which included falls, exposure to mechanical forces, traffic injuries, interpersonal violence, animal contact, foreign bodies, and self-harm.

Fourth, the Slope Index of Inequality (SII) and Concentration Index (CI) were calculated as standardized metrics to quantitatively assess inequalities in the incidence, prevalence, and YLDs of nerve injury at the global level and within the 5 SDI regions (17).

Fifth, to provide evidence for targeted prevention strategies, we comprehensively analyzed, characterized, and predicted trends in the incidence, prevalence, and YLDs of nerve injury on a global scale.

Sixth, a Shapiro–Wilk test confirmed the non-normal distribution of the data (*p* < 0.001). We therefore incorporated the SDI, a composite indicator integrating per capita income, years of education, and fertility rate, into our analysis. In the GBD 2021 study, the SDI ranges from 0 to 1, where 0 represents the lowest per capita income, the fewest years of schooling, and the highest fertility rate, and 1 represents the opposite (7). Spearman correlation analysis was used to examine the association between SDI and age-standardized YLD rates for 204 countries and territories in 2021 (18, 19).

All statistical analyses and graphing were performed using R software (Version 4.1.3) and GraphPad Prism (Version 7.0.0). A two-tailed *p*-value < 0.05 was considered statistically significant.

## 3 Result

### 3.1 Global and regional level

In 2021, there were 4.13 million (95% UI: 3.11–5.56) cases of neurological injury worldwide, with an age-standardized incidence of 53 cases per 100,000 people (95% UI: 40–72). From 1990 to 2021, there was a slight decline in age-standardized incidence worldwide [Average Annual Percent Change (AAPC): −1.03, 95% UI: −1.38 − 0.68, *p* < 0.01]. In terms of SDI regions, the middle SDI region witnessed the highest number of cases, reaching 1.11 million (95% UI: 0.81–1.54). However, the highest incidence of ASR (per 100,000 people) was found in areas with high SDI [75.46 (95% UI: 53.6–88.92)]. Looking dynamically at the prevalence of nerve injury by SDI region from 1990 to 2021, AAPC exhibited a downward trend in all regions by region, with South Asia having the highest number of cases in 2021 at 750,000 (95% UI: 0.52–1.04). With age standardization, incidence varies by region. The top 3 regions with ASR incidence (per 100,000 people) above 100 are Australasia [169.16 (95% UI: 110.88–257.11)] and Eastern Europe [145.61(95% UI: 101.23–208.27)] and Latin America South [131.18 (95% UI: 87.7–195.96)]. West sub-Saharan Africa [34 (95% UI: 27–46)], Central sub-Saharan Africa [35.88 (95% UI: 27.58–47.43)] and sub-Saharan Africa [37.1 (95% UI: 28.6–48.49)] had the lowest age standardization rate. When researchers took a dynamic perspective on the age-standardized rate (ASR) of nerve injury incidence across different regions during the period from 1990 to 2021, AAPC showed a decreasing trend in nearly 90% of regions, with Eastern sub-Saharan Africa [−3.26 (95% UI: −7.06−−0.71), *p* = 0.11], Andean Latin America [−1.33 (95% UI: −1.52 − 1.14), *p* < 0.01], and high-income North America [−1.31% (95% UI: −1.56 − 1.06), *p* < 0.01] being the three regions where AAPC declined by more than 1.3. However, there is still one region, the Caribbean [AAPC: 0.25% (95% UI: 0.14–0.65), *p* = 0.20], where age-standardized incidence is continuing to rise (Supplementary Table S1).

In 2021, the number of cases of neurological injury is 4.27 million (95% UI: 3.4–4.75) and the age-standardized prevalence is 51 cases per 100,000 population (95% UI: 47–57). From 1990 to 2021, the global age-standardized prevalence showed a slight decline (AAPC: −0.99, 95% UI: −1.06 − 0.93, *p* < 0.01). The area with the highest prevalence of SDI was high SDI [1.32 million (95% UI: 1.22–1.42)], and the incidence of ASR (per 100,000 people) was consistent with the prevalence. The incidence trend in 5 SDI regions was still decreasing. South and East Asia stand out as the two regions boasting the highest patient numbers. When it comes to age-standardized prevalence, 50% of all regions surpassed the global average. The top two regions with a neurological injury prevalence of more than 100 were Australasia [185.86 (95% UI: 168.13–210.1)] and Southern Latin America [156.81 (95% UI: 142.16–173.66)]. Prevalence of ASR below 35 is concentrated in central sub-Saharan Africa, East Asia, Southeast Asia, and sub-Saharan Africa, with the lowest prevalence of neurological injury in western sub-Saharan Africa [27.22 (95% UI: 24.42–30.91)]. The AAPC, which dynamically looked at the prevalence of nerve injury by region from 1990 to 2021, showed a downward trend in most regions. Two Asian regions, Central Latin America [AAPC: 1.23% (95% UI: 1.38–1.09), *p* < 0.01] and Southern Sub-Saharan Africa [AAPC: 1.23% (95% UI: 1.31–1.15), *p* < 0.01], had the highest rates of decline. However, two regions, the Caribbean [AAPC: 0.61% (95% UI: 0.34–0.87), *p* < 0.01] and Oceania [AAPC: 0.57% (95% UI: 0.43–0.71), *p* < 0.01], showed the fastest increase in prevalence (Supplementary Table S1).

In 2021, penetration injuries resulted in 440,000 (95% UI: 0.3–0.6) YLDs, corresponding to an age-standardized rate of 5.26 cases per 100,000 population (95% UI: 3.63–7.2). From 1990 to 2021, the global age-standardized YLDs showed a slight decline (AAPC: −1.01, 95% UI: −1.06 – 0.95). The change of YLDs in the 5 SDI regions was highly consistent with the prevalence rate, and the highest was in the high SDI region [130,000 (95% UI: 0.09–0.18)]. When divided by 21 GBD regions based on the age-standardized YLDs, South Asia and East Asia remain the two regions with the highest YLDs. The pattern of age-standardized YLDs shows a remarkable similarity to that of age-standardized prevalence. The two regions with the highest incidence of neurologically impaired YLDs in ASR remain Australasia [19.32 (95% UI: 12.96–26.67)] and Southern Latin America [16.39 (95% UI: 10.97–22.43)]. Western sub-Saharan Africa [2.82 (95%UI: 1.99–3.77)] and Central sub-Saharan Africa [3.38 (95%UI: 2.33–4.93)] had the lowest incidence of neuro-damaging YLDs in ASR. Similarly, we estimated the AAPC of YLDs under age standardization from 1990 to 2021, with 2 regions showing an upward trend. It remains the Caribbean [AAPC: 0.57% (95% UI: 0.37–0.77), *p* < 0.01] and Oceania [AAPC: 0.58% (95% UI: 0.53–0.63), *p* < 0.01]. Southern Sub-Saharan Africa [AAPC: −1.26% (95% UI: −1.33 − 1.19), *p* < 0.01] and Central Latin America [AAPC: −1.23% (95% UI: −1.38 − 1.08), *p* < 0.01] are the regions with the fastest declines (Supplementary Table S1).

### 3.2 National levels

**Figure 1:**
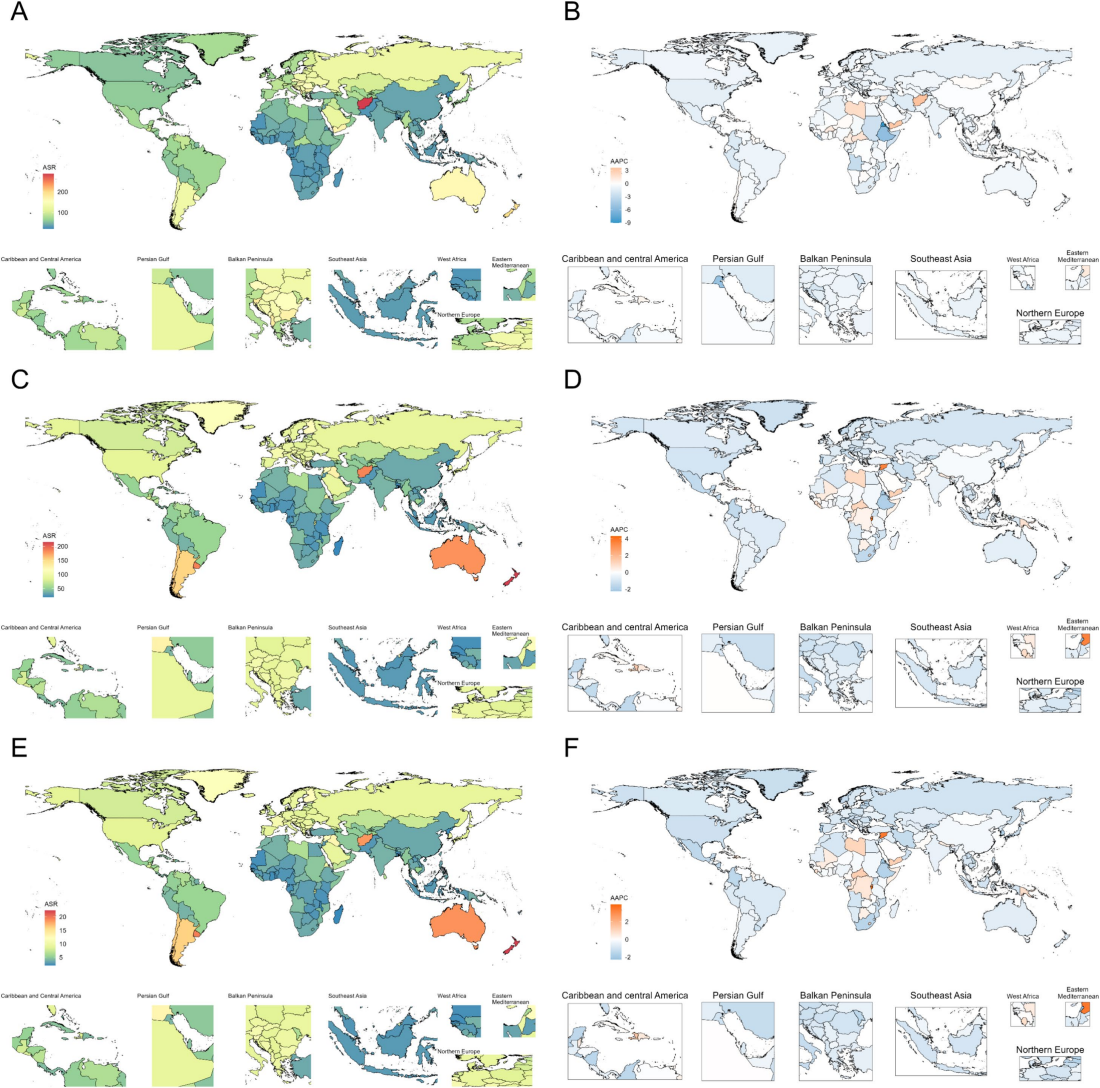
In 2021, the global map presents the age-standardized rates per 100,000 persons of nerve injury for incidence **(A)**, prevalence **(C)**, and YLDs **(E)**, as well as the rates of global mean annual percentage change (AAPC) for incidence **(B)**, prevalence **(D)**, and YLDs **(F)** of nerve injury.

Global maps of nerve injury ASIR reveal substantial geographical disparities. For instance, Afghanistan exhibits a relatively high ASIR, whereas incidence varies markedly across other regions. Additionally, the global spatial distribution of AAPC trends for nerve injury ASIR shows significant regional heterogeneity (Figure 1).

### 3.3 Global and different SDI level regions

**Figure 2:**
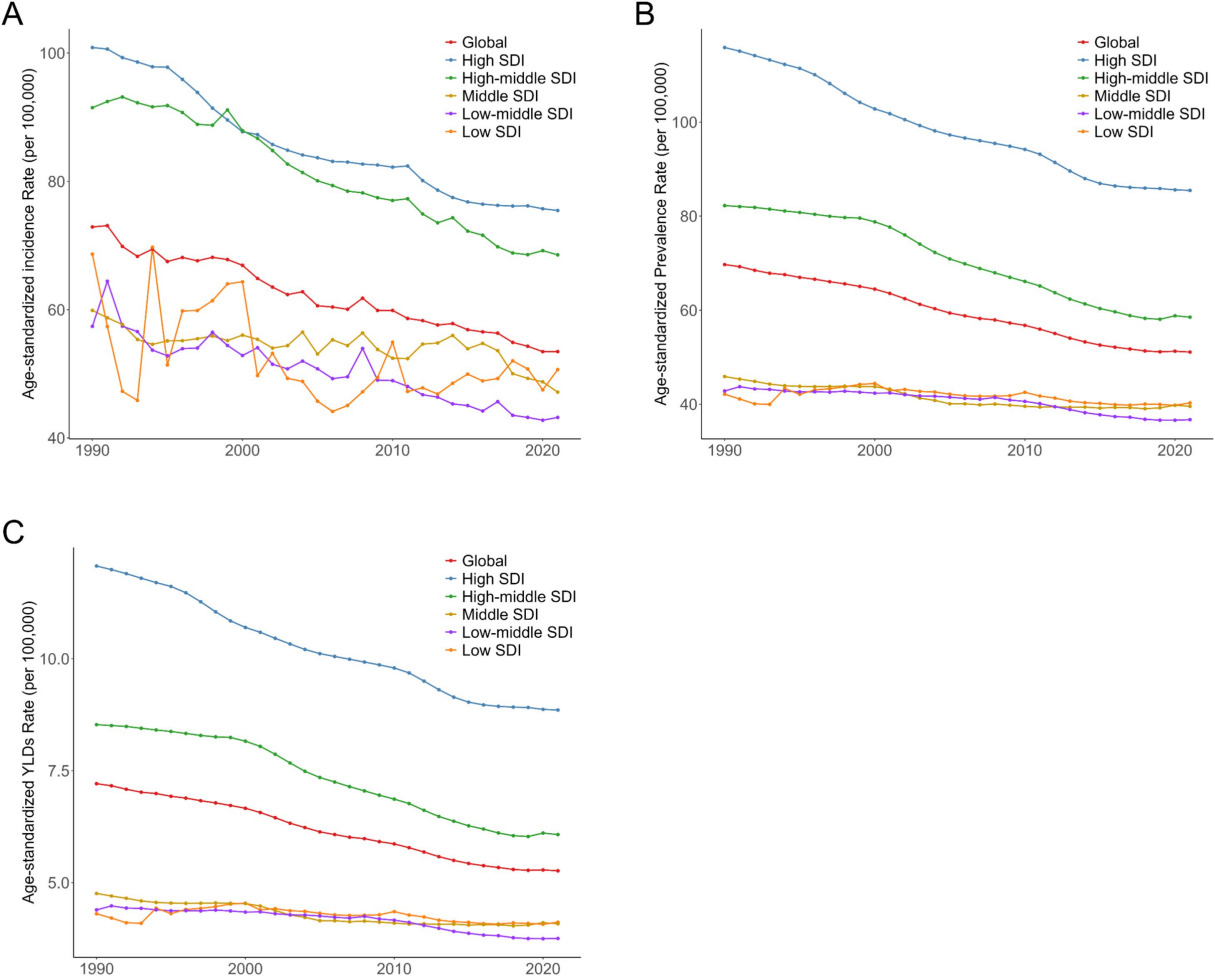
Trends in age-standardized incidence **(A)**, prevalence **(B)**, and YLDs **(C)** of nerve injury globally and in 5 SDI regions from 1990 to 2021.

The global trend in ASIR was similar to that of high and high-middle SDI regions, with all showing a downward trajectory. However, the global ASIR (approximately 55 per 100,000 population in 2021) was slightly lower than that of these two regions (Figure 2A). High SDI regions had a high baseline ASIR (nearly 100 per 100,000 population in 1990) followed by a steady, continuous decline, reaching approximately 75 per 100,000 population by 2021. High-middle SDI regions also had a high 1990 ASIR (slightly lower than high SDI regions) with a gradual but fluctuating decline, reaching approximately 60 per 100,000 population by 2021.

Middle, low-middle, and low SDI regions had relatively low 1990 ASIRs with significant fluctuations over the three decades. Middle and low-middle SDI regions showed a distinct upward trend in the latter part of the study period, while low SDI regions also experienced an increase around 2021,though their ASIR remained lower than other regions. From 1990 to 2021, trends in prevalence and YLDs were consistent with incidence (Figure 2).

### 3.4 National level

**Figure 3:**
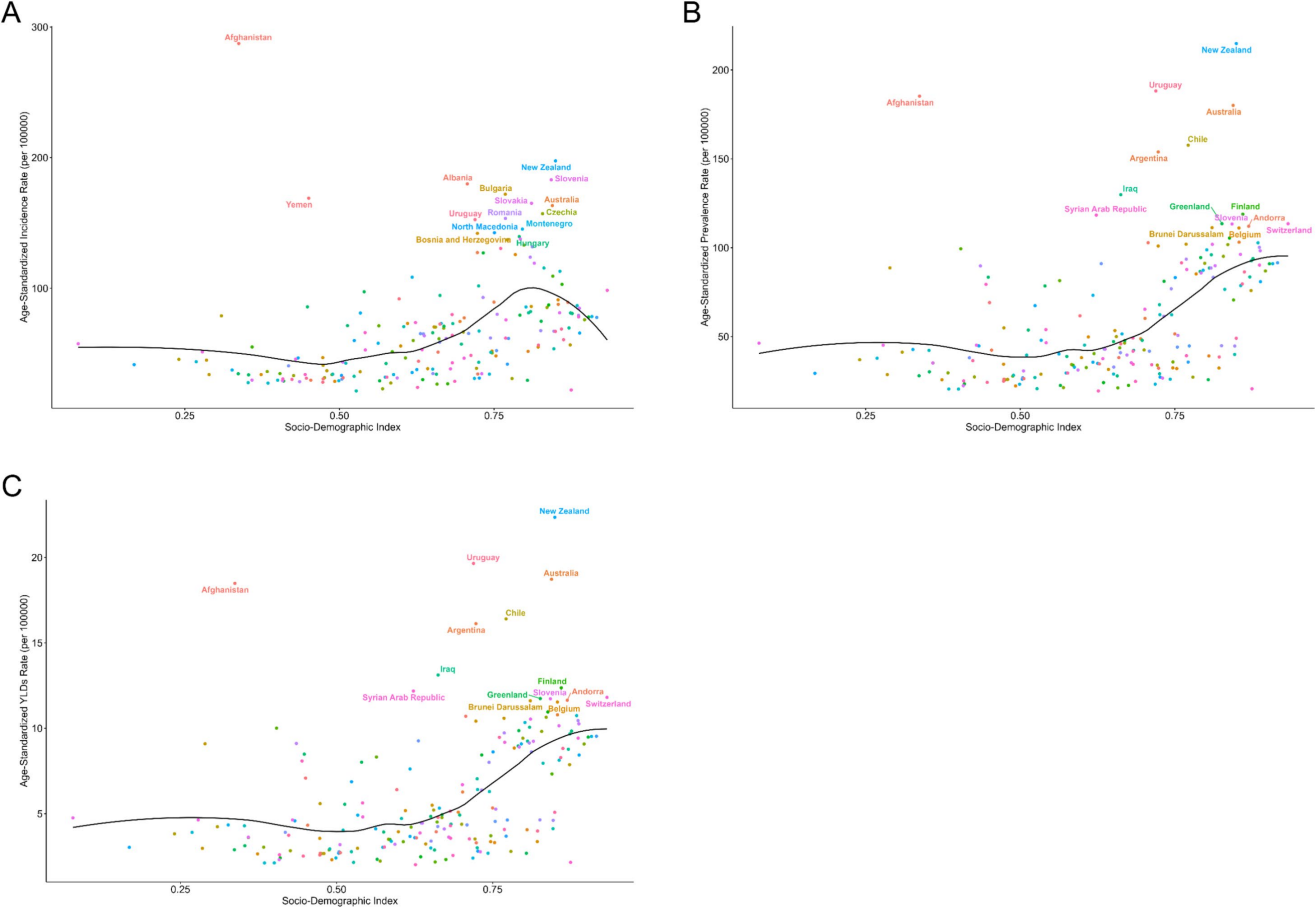
Age-standardized incidence **(A)**, prevalence **(B)**, and YLDs **(C)** of nerve injury in 204 countries.

The curve depicting the relationship between national SDI and ASIR follows an inverted U-shape: (1) at low SDI (<0.5), the curve is relatively flat, indicating a low and stable ASIR; (2) as SDI increases to 0.5–0.75, the curve rises, reflecting a gradual increase in ASIR; (3) when SDI exceeds 0.75, the curve declines, with ASIR beginning to decrease (Figure 3A). The incidence varies widely among countries. Among them, Afghanistan and Yemen have low SDI but high incidence; New Zealand and Australia have a high SDI and a moderate incidence, while other countries such as Albania, Bulgaria and Slovenia are distributed in different locations, reflecting their different incidence and SDI situation. As can be seen from the figure, age-standardized prevalence and YLDs are roughly the same as the age-standardized incidence distribution (Figure 3).

### 3.5 Causes of nerve injury

**Figure 4:**
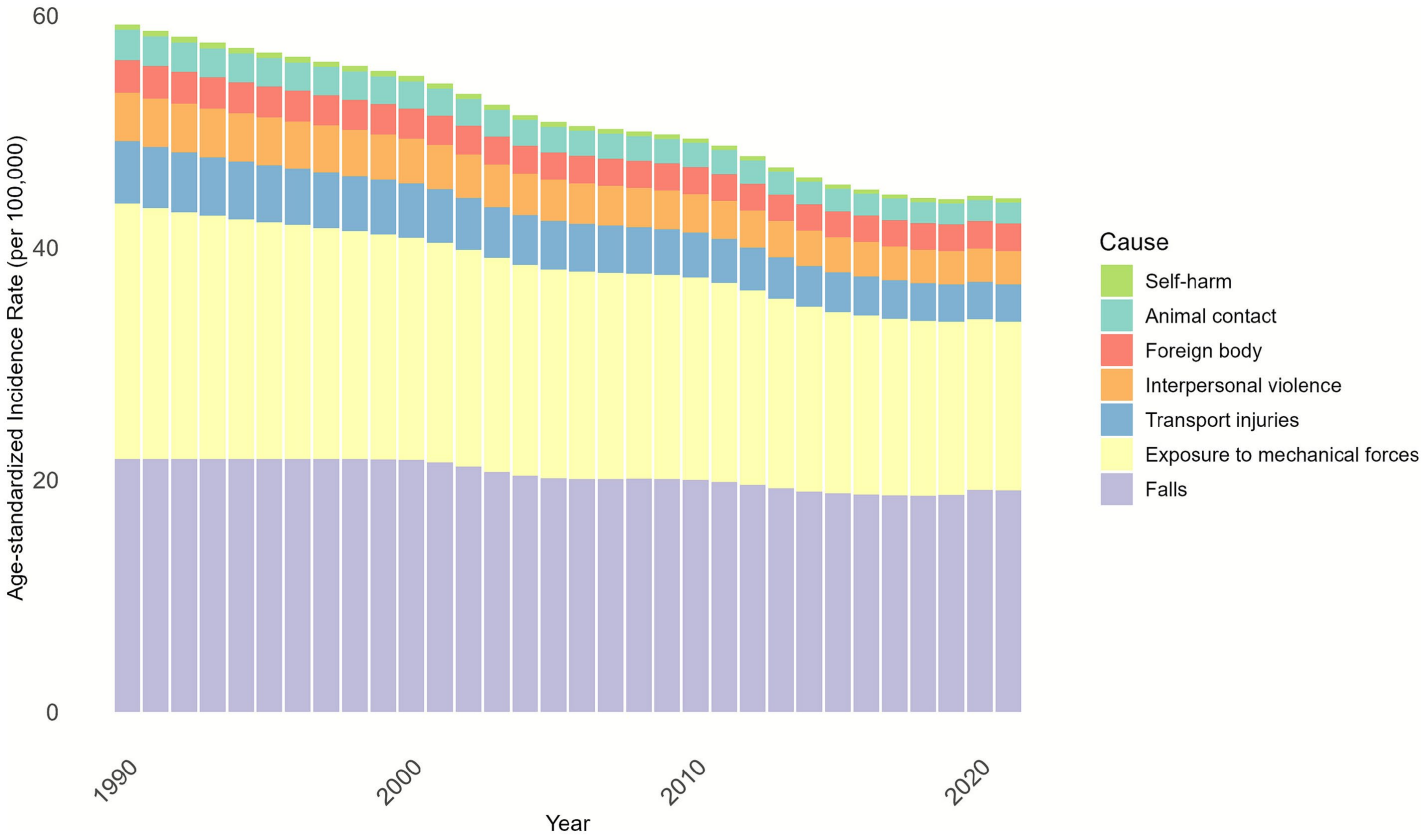
Cause composition of age-standardized Incidence of nerve injury by GBD for both males and females from 1990 to 2021.

Globally, the leading causes of nerve injury (per GBD etiological classification) were falls, exposure to mechanical forces, and road traffic injuries, with falls accounting for over 40% of all cases (Figure 4).

**Figure 5:**
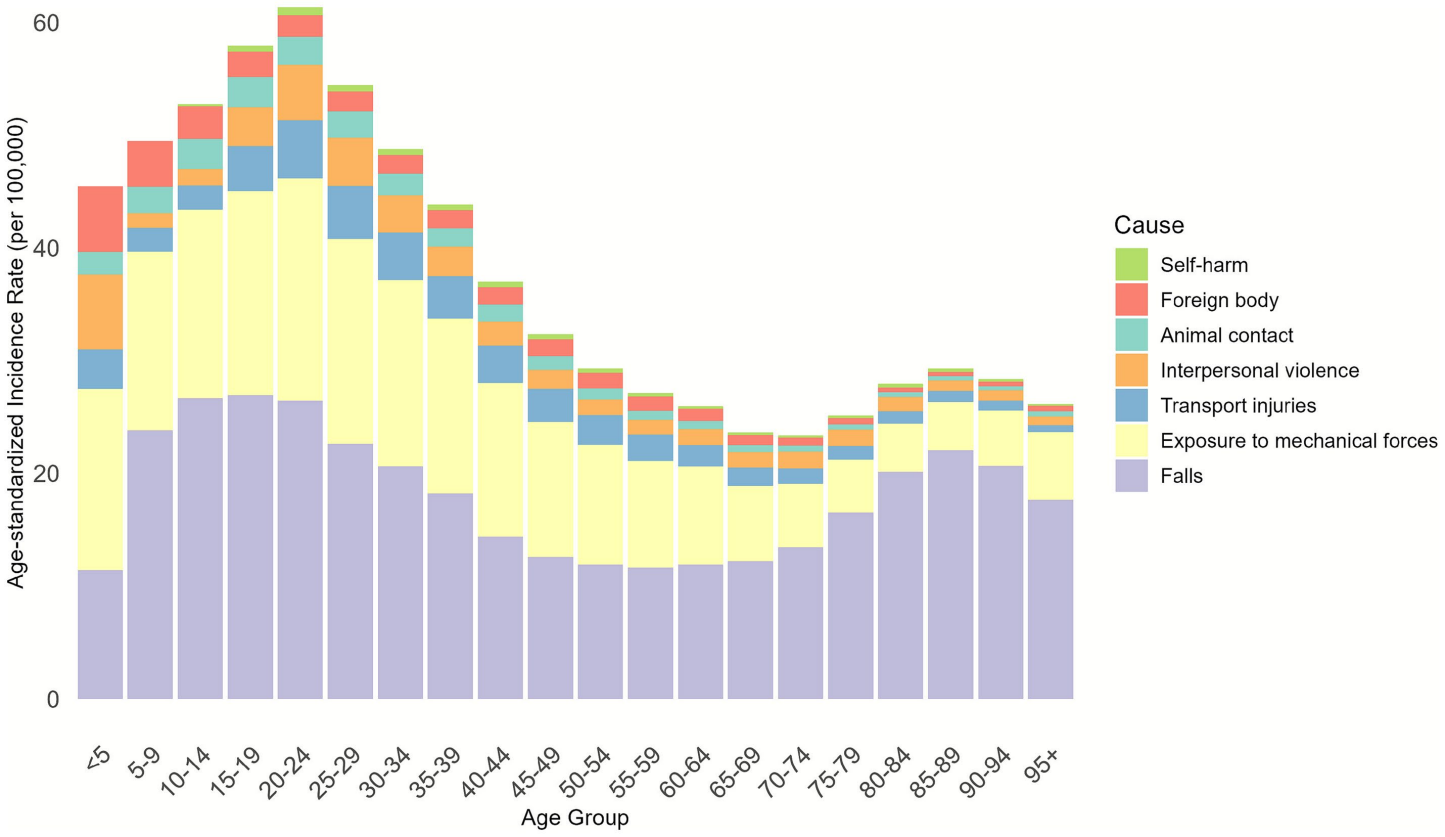
Cause composition of age-standardized incidence of nerve injury for both males and females in different age groups in 2021.

We further conducted an analysis on the distribution of injury causes across different age groups and discovered that falls and exposure to mechanical forces were the primary causes of injuries in all age groups. The relatively higher overall incidence observed in the 10–34-year age group can be attributed to exposure to mechanical forces. For individuals aged ≥35 years, falls became the predominant cause, with the proportion of fall-related nerve injury increasing with age, accounting for nearly 80% of cases in the ≥85-year age group (Figure 5).

From a compositional perspective, falls are the predominant cause of nerve injury, with their composition ratio rising steadily from approximately 35% in 1990 to around 45% in 2020. The second most common cause is “exposure to mechanical forces,” accounting for approximately 30 to 35%. In contrast, the composition ratios of traffic injuries, interpersonal violence, foreign bodies, animal contact, and self-harm are relatively stable and account for only a small proportion (Supplementary Figure S7).

### 3.6 Age and sex level

Observing the incidence trends of each age group and gender in 2021, we found that the incidence among males increases in the age group of 5–19 years, decreases rapidly from the age group of 20–24 years to the age group of 75–79 years, and increases rapidly and slightly from the age group of 76 to 79 years to the age group of 85 to 89 years. There was a slight decline in the age group of 86–89 years. In contrast, the incidence among women manifested a gradual decrease prior to and within the 55–59 age group, subsequently followed by a gradual rise from the 60–69 age group to the 85–89 age group, but at a faster rate than in men of the same age group. In the 86–89 age group, the incidence declined in a similar way to that of males, and the incidence among males peaked between 15 and 19 years of age. In addition, at age 75 and beyond, the incidence is higher in women than in men (Supplementary Figure S1a).

We also looked at trends in age-standardized incidence in men and women from 1990 to 2021. In general, the fluctuation is not significant, showing a slight downward trend, and the downward trend of men is greater than that of women. From the perspective of the number of cases, taking the age group of 15–19 years old as an example, the number of males exceeds 300,000, which is at a relatively high level, while the number of females is about 200,000, which is relatively low. Overall, the number of cases in both men and women declined with age, with a more significant decrease at higher ages. From 1990 to 2021, the number of male cases was slightly higher than that of female cases, and both numbers remained relatively stable with no significant fluctuations. For example, in 1990, the number of men and the number of women were both at a high level, and the number has changed slightly from year to year since then, but has remained roughly within a certain range (Supplementary Figure S2a).

### 3.7 Decomposition analysis

Three key factors contributed to global nerve injury burden trends: population growth, epidemiological changes, and population aging. Globally, population growth had the strongest correlation with incidence. Population aging was generally positively associated with incidence, while epidemiological changes showed a negative association. However, the contribution of each factor varied by region and deviated from the global pattern.

Population growth had a consistent impact across all five SDI regions (aligning with the global trend). Notably, in high-middle and low SDI regions, population aging was associated with a reduction in nerve injury incidence—contrary to the global positive correlation (Supplementary Figure S3a).

### 3.8 Prediction of global nerve injury incidence

Supplementary Figure S4a shows a gradual decline in the global ASIR of nerve injury from 1990 to 2021, indicating an overall improvement in nerve injury prevention over the three decades. Using the Bayesian adaptive prediction curve (BAPC) model, we projected the age-standardized incidence rate from 2020 to 2030. The trend suggests a continued decline or stabilization; however, prediction uncertainty increases as the time horizon approaches 2030.

### 3.9 Inequality analysis

Nerve injury incidence showed a distinct distribution pattern across SDI levels, with incidence changing dynamically as SDI varies (Supplementary Figure S5). In 2021, for the same cumulative population proportion, the cumulative incidence rate was higher than in 1990, suggesting either an increase in overall incidence or a more uneven distribution.

The concentration index (CI) for 1990 was −0.033 (close to 0), indicating a relatively balanced distribution of nerve injury incidence across SDI groups with no significant concentration in high- or low-SDI populations. The 2021 CI was 0.021, a small positive value suggesting a slight trend toward concentration of nerve injury in high-SDI populations. However, this trend lacks statistical significance, as the 95% confidence interval includes 0 (Supplementary Figure S6a).

## 4 Discussion

In this study, we analyzed the incidence, prevalence, and YLDs of nerve injury using GBD 2021 data spanning from 1990 to 2021. The objective was to characterize, model, and compare the epidemiological patterns of nerve injury across age groups, genders, and etiological mechanisms at the global, regional, and national levels, thereby systematically quantifying its disease burden. A growing body of evidence highlights that non-fatal health losses attributable to nerve injury constitute a substantial component of the global injury burden, insights that are critical for policymakers to optimize the allocation of limited healthcare resources and develop targeted prevention and management strategies.

A comprehensive look at global nerve injury epidemiology data from the GBD database shows that the regions with the highest incidence, prevalence, and YLDs in 2021 are predominantly located in South and East Asia. We also report that despite the overall downward trend in global incidence, the incidence rates are increasing in the Caribbean and Oceania. The age-specific prevalence and AAPC of YLDs in these two regions also showed the most rapid increase during the same period. By region, the regions with the highest age-standardized incidence, prevalence, and YLDs in 2021 are Australasia and southern Latin America. South and East Asia are among the most densely populated regions globally, and the large population base results in a high absolute number of patients with nerve injury, resulting in statistically high levels of incidence, prevalence, and YLDs. Some countries in the Caribbean and Oceania are experiencing demographic changes and increasing aging. With the increase of age, the function of the nervous system of the human body gradually declines, and the risk of neurodegenerative diseases (such as Alzheimer’s disease, Parkinson’s disease, etc.) and cerebrovascular diseases increases, leading to nerve injury, thus increasing the incidence, prevalence and YLDs of nerve injury (20). This age-related vulnerability underscores the need for region-specific interventions tailored to aging populations.

To inform targeted prevention, we analyzed age-stratified etiologies of nerve injury from the GBD database. Falls, exposure to mechanical forces, and road traffic injuries emerged as the leading causes. Specifically, the 10–34-year age group, characterized by high physical activity, is disproportionately at risk of nerve injury due to occupational and recreational exposure to mechanical forces. While mechanical force-related nerve injury was the primary cause between 1990 and 2012, its contribution gradually declined over time. In contrast, falls became increasingly dominant with age: among individuals aged 55–59 years and older, falls accounted for over 50% of nerve injuries, and nearly 80% of nerve injuries in the ≥85-year age group were fall-related. Notably, from a compositional standpoint, falls have increasingly dominated the incidence, prevalence, and disability burden of nerve injuries between 1990 and 2020, emerging as a key target for prevention and intervention in the field of public health. Falls are well-documented as a major public health concern among older adults (21, 22), emphasizing the urgency of mitigating fall-related nerve damage in this population. A review of relevant literature suggests that geriatric-targeted interventions can effectively reduce both fall risk and subsequent nerve injury. From a preventive standpoint, enhancing balance and physical fitness is foundational: virtual reality-based balance training has been shown to reduce fall incidence in older adults (23). Environmental modifications tailored to elderly mobility patterns—such as installing safety railings on home stairs and improving shock absorption in high-activity areas, can further minimize injury severity during falls. Additionally, wearable technologies (e.g., sensor-integrated seatbelts or airbag-equipped jackets) offer promising avenues to cushion impact forces and prevent nerve trauma (24). While these strategies hold significant potential, their long-term efficacy and scalability require further validation.

As illustrated in Figure 2, the age-standardized incidence of nerve injury showed a downward trend globally and in high Socio-demographic Index (SDI) regions, likely reflecting improvements in healthcare access, living standards, and injury prevention infrastructure in these settings. In contrast, middle and low SDI regions exhibited greater incidence fluctuations and an overall increasing trend, which may stem from unmet healthcare needs, inadequate public health systems, and limited injury prevention resources. These disparities underscore the importance of developing region-specific public health policies that account for local epidemiological characteristics and resource constraints (25, 26).

When we examined the incidence by age group, we discovered that the incidence of nerve injury in 2021 for both the overall population and men reached its peak in the 15–19 year age group, subsequently, there was an overall downward trend, and the disparity decreases as age increases. These situations remind us to increase the investment and allocation of health resources, as well as to strengthen the protection of young and old people. Although more and more research is now highlighting the significance of youth health for global development (27), progress is not fast and there are still great difficulties in making young people better (28). Trend analysis of gender-specific incidence and case numbers showed relatively stable case counts for both sexes over time, alongside a gradual decline in age-standardized incidence. This reduction may reflect incremental successes in nerve injury prevention, diagnosis, and management. Gender-stratified comparisons provide a basis for analyzing sex-specific disparities and their temporal evolution, enabling the development of gender-responsive intervention strategies.

Over the past three decades, changes in nerve injury incidence, prevalence, and YLDs have been primarily driven by population aging, epidemiological transitions, and demographic shifts. Globally, population size changes exerted a significant influence on overall nerve injury burden, with epidemiological transitions also playing a pivotal role; aging, while a contributing factor, was not the primary determinant. For males, nerve injury incidence was closely associated with population size and epidemiological factors. In contrast, aging accounted for a larger proportion of nerve injury burden in females, possibly due to sex-specific physiological vulnerabilities and longer life expectancy. The relative contribution of these factors varied across SDI regions, reflecting differences in economic development, lifestyle patterns, healthcare resource availability, and injury prevention capacity. For example, high SDI regions may benefit from advanced fall prevention programs for the elderly, while low SDI regions may face greater challenges from occupational mechanical injuries due to limited workplace safety regulations.

Inequality analysis demonstrated that, compared with 1990, the cumulative incidence of nerve injury for the same cumulative population proportion was higher in 2021. This increase may be attributed to a combination of epidemiological changes, population growth, environmental and lifestyle shifts, and advancements in medical diagnostic technologies (which improve case detection). Additionally, uneven distribution of nerve injury risk, driven by inadequate healthcare and health education in low SDI regions and high work-related stress in high SDI regions, may have exacerbated this trend. The concentration index (CI) for 1990 was close to 0, indicating a relatively equitable distribution of nerve injury risk across social strata. This balance may have been facilitated by more uniform social environments, similar lifestyle patterns, and standardized public health interventions at the time. In 2021, the CI was slightly positive but not statistically significant (95% confidence interval included 0), suggesting a tentative trend toward concentration of nerve injury in high SDI populations, potentially due to elevated risk from fast-paced lifestyles and high work intensity. Confirmation of this trend will require larger sample sizes and more in-depth analyses; if validated, interventions should target high SDI populations while ensuring continued attention to nerve injury prevention in low SDI regions to avoid widening health disparities.

In conclusion, nerve injury remains a major global health concern, with persistently high incidence rates and rising burden in the Caribbean and Oceania. Falls and exposure to mechanical forces are the dominant etiological factors, and population growth and epidemiological transitions are the primary drivers of burden changes. Adolescents and older adults are at particular risk, and gender- and region-specific disparities in burden underscore the need for targeted interventions. Against the backdrop of global population aging and sustained population growth, reducing nerve injury incidence is an urgent priority. Targeted etiological analysis and the implementation of comprehensive prevention measures, such as geriatric fall prevention programs, adolescent injury education, and workplace safety initiatives, have the potential to significantly reduce casualties and alleviate disease burden. Achieving long-term prevention goals will require enhanced social support, improved infrastructure, and equitable access to healthcare resources across all SDI regions.

## 5 Strengths and limitations

The study has some merits. First, it provides an updated global, regional, and national burden of nerve injury from 1990 to 2021. These research outcomes can assist policymakers in acquiring the latest epidemiological characteristics of nerve injury in 5 SDI regions and 21 GBD regions. Second, the 21 regional differences when compared with the five SDI regions can provide richer information and help policymakers determine which factors should be focused on to enhance the efficacy of preventive interventions.

There are certain drawbacks to this study. Firstly, the study might also underestimate the actual prevalence. The GBD 2021 study computed the incidence of non-fatal injuries based on the probability of permanent health loss (9). Permanent health loss is related to the probability that the patient (1 year after getting injured) will return to a disabled state that is worse than their pre-injury health condition (9). Using the GBD data, we attempted to figure out the lifetime prevalence of nerve injury but failed. The reason is that although longitudinal cohort or survey analyses are frequently employed to calculate lifetime prevalence, GBD studies are cross-sectional analyses that are carried out on an annual basis. In the GBD study, it is presumed that the period of short-term disability resulting from non-fatal injuries is shorter than 1 year. This implies that it may not be feasible to calculate the prevalence of future rounds for the short-term disability following nerve injury in a specific year (9). Secondly, a drawback of this study is that it lacks the capacity to precisely estimate the incidence within countries with relatively poor-quality data. For some countries with scanty data, their estimates are modeled and projected by adopting the estimates from countries with plentiful data (9). Consequently, the estimates for countries with insufficient data (e.g., the Democratic People’s Republic of Korea) might deviate more from the true incidence. Thirdly, the GBD 2019 study did not connect nerve injury with risk factors like behavioral aspects (e.g., alcohol consumption), so we could not evaluate any correlation between the two (e.g., how much disease burden was brought about by certain risk factors) (29). Fourthly, the specific data of 204 countries were not analyzed.

## 6 Implications for future studies

First, population-based epidemiological studies could be enhanced in the future by making definitions more standardized (e.g., having standardized diagnostic criteria), by improving methodologies (e.g., determining cases based on multiple overlapping information sources like hospitals, outpatient clinics, general practitioners, and death certificates), and by optimizing data presentation (e.g., collecting comprehensive data for a whole calendar year). These enhancements are especially valuable in places where data is in short supply and can assist in making it easier to compare different countries. Second, there’s a need for more effective methods to describe the risk of long-term disabling sequelae that is currently not fully recognized, so as to make the estimations closer to the real period prevalence. Besides, we hope that the GBD collaborators will incorporate lifetime prevalence in their future rounds of work to precisely evaluate the global influence of nerve injury. Finally, we would like the GBD collaborators to include the burden of nerve injury that can be traced back to risk factors. This will offer more detailed outcomes regarding the causes of nerve damage and enable the formulation of more targeted prevention strategies.

## 7 Conclusion

In summary, the global age-standardized incidence of nerve injury has exhibited a consistent downward trend since 1990, with the Caribbean and Oceania being the only two regions where incidence has continued to rise. Against the backdrop of global population aging and sustained demographic growth, reducing the incidence of nerve injury remains an urgent public health priority. Consequently, targeted investigation of nerve injury risk factors and the implementation of comprehensive preventive strategies have substantial potential to reduce associated morbidity and mitigate the global disease burden (30, 31). Furthermore, the successful execution of effective long-term prevention initiatives will require strengthened social support systems, upgraded public health infrastructure, and equitable allocation of healthcare resources across regions with varying SDI levels.

## Data Availability

The original contributions presented in the study are included in the article/Supplementary material, further inquiries can be directed to the corresponding authors.
