# Supplementary material for: A burden of nerve injury from a global perspective, 1990–2021: an analysis of incidence, prevalence, and years lived with disability
Source: Front Neurol. 2026 Jan 6;16:1669662. doi: 10.3389/fneur.2025.1669662 (PMC12815850; doi:10.3389/fneur.2025.1669662)
Supplement: Supplementary file 1 [file Data_Sheet_1.PDF]

Table.S1 The incidence, prevalence, YLDs of nerve injury in 2021, and the percentage changes in age - standardized rates in various regions in 2021

YLDs: years lived with disability; GBD: Global Burden of Diseases, Injuries, and Risk Factors; UI, uncertainty interval.

Location	Sex	Incidence				Prevalence				YLDs			
		Number in 2021 (95%UI)	ASR in 2021 (per 100,000, 95%UI)	Average Annual Percent Change (95% CI)	P-values	Number in 2021 (95%UI)	ASR in 2021 (per 100,000, 95%UI)	Average Annual Percent Change (95% CI)	P-values	Number in 2021 (95%UI)	ASR in 2021 (per 100,000, 95%UI)	Average Annual Percent Change (95% CI)	P-values
Global	Both	4134699 (3111514,5559138)	53.46 (40.17,71.92)	-1.03 (-1.38 - -0.68)	0.00	4274048 (3899941,4747060)	51.11 (46.58,56.84)	-0.99 (-1.06 - -0.93)	0.00	440250 (303895,601450)	5.26 (3.63,7.2)	-1.01 (-1.06 - -0.95)	0.00
	Female	1505212 (1051897,2178714)	39.59 (27.83,57.22)	-0.85 (-0.92 - -0.78)	0.00	1742688 (1571154,1933977)	40.23 (36.17,44.79)	-0.80 (-0.90 - -0.71)	0.00	177491 (120934,242599)	4.11 (2.79,5.62)	-0.82 (-0.90 - -0.74)	0.00
	Male	2629487 (2024772,3488576)	66.9 (51.56,88.92)	-1.13 (-1.36 - -0.90)	0.00	2531360 (2313499,2811341)	62.25 (56.85,69.35)	-1.12 (-1.20 - -1.05)	0.00	262759 (181855,357149)	6.45 (4.46,8.77)	-1.13 (-1.19 - -1.07)	0.00
High SDI	Both	716174 (516350,988649)	75.46 (53.6,104.49)	-1.00 (-1.18 - -0.83)	0.00	1320995 (1221320,1424672)	85.48 (78.21,93.4)	-1.09 (-1.17 - -1.01)	0.00	135057 (91993,182459)	8.85 (5.97,12.02)	-1.09 (-1.17 - -1.00)	0.00
	Female	263458 (180457,383490)	55.84 (38.64,81.02)	-0.60 (-0.79 - -0.40)	0.00	552057 (507200,596643)	66.59 (60.71,72.97)	-0.77 (-0.88 - -0.66)	0.00	55758 (38363,75500)	6.84 (4.58,9.33)	-0.77 (-0.88 - -0.65)	0.00
	Male	452716 (331817,618636)	93.91 (68.45,129.09)	-1.20 (-1.38 - -1.03)	0.00	768938 (712347,832996)	104.44 (95.56,114.81)	-1.32 (-1.41 - -1.24)	0.00	79299 (53838,107510)	10.86 (7.4,14.7)	-1.32 (-1.40 - -1.23)	0.00
High-middle SDI	Both	794481 (567608,1105037)	68.54 (49.63,94.88)	-0.93 (-1.01 - -0.85)	0.00	970794 (892357,1057624)	58.52 (53.15,64.76)	-0.98 (-1.01 - -0.96)	0.00	100291 (67562,137012)	6.07 (4.03,8.33)	-1.00 (-1.03 - -0.98)	0.00
	Female	264033 (178880,393991)	47.8 (32.48,71.27)	-0.60 (-0.73 - -0.48)	0.00	375212 (340215,412853)	43.26 (38.69,48.38)	-0.64 (-0.70 - -0.59)	0.00	38346 (25521,52591)	4.46 (2.92,6.22)	-0.67 (-0.74 - -0.60)	0.00
	Male	530448 (387295,712623)	87.93 (64.75,118.51)	-1.12 (-1.23 - -1.01)	0.00	595582 (546627,650494)	74.44 (67.59,82.09)	-1.24 (-1.29 - -1.19)	0.00	61945 (41987,84815)	7.75 (5.19,10.58)	-1.25 (-1.29 - -1.21)	0.00
Low SDI	Both	644240 (462454,929319)	50.66 (36.74,71.93)	-1.02 (-1.15 - -0.89)	0.39	322029 (253384,446305)	40.29 (32.55,55.09)	-0.56 (-0.64 - -0.47)	0.17	33348 (23171,48585)	4.12 (2.91,5.97)	-0.57 (-0.65 - -0.49)	0.22
	Female	230643 (159788,327361)	36.81 (25.39,52.14)	-1.11 (-1.31 - -0.91)	0.00	123923 (101071,165626)	32.09 (26.6,42.74)	-0.59 (-0.72 - -0.46)	0.05	12732 (8666,18382)	3.24 (2.23,4.64)	-0.60 (-0.73 - -0.48)	0.04
	Male	413597 (293449,600334)	64.59 (46.55,92.95)	-0.94 (-1.03 - -0.84)	0.39	198106 (150238,278192)	48.55 (38.13,65.86)	-0.51 (-0.59 - -0.44)	0.19	20616 (14033,31024)	5 (3.51,7.38)	-0.51 (-0.58 - -0.45)	0.27
Low-middle SDI	Both	861005 (643939,1146703)	43.2 (32.32,57.54)	-0.97 (-3.13 - 1.24)	0.00	613777 (546208,706069)	36.72 (32.95,41.62)	-0.21 (-0.51 - 0.09)	0.00	63343 (43918,86973)	3.75 (2.61,5.12)	-0.20 (-0.52 - 0.12)	0.00

	Female	343054 (234459,501050)	35.2 (24.14,51.23)	-0.72 (-1.09 - -0.35)	0.00	272356 (237222,314073)	32.73 (28.39,37.48)	-0.16 (-0.33 - 0.00)	0.00	27775 (18567,38632)	3.3 (2.23,4.55)	-0.17 (-0.33 - -0.01)	0.00
	Male	517951 (401947,679031)	51.05 (39.63,66.79)	-0.95 (-3.08 - 1.23)	0.00	341422 (303050,393572)	40.55 (36.32,46.21)	-0.17 (-0.44 - 0.09)	0.00	35568 (24478,48705)	4.19 (2.92,5.72)	-0.16 (-0.45 - 0.13)	0.00
Middle SDI	Both	1114121 (811970,1542266)	47.14 (34.46,65.16)	-0.80 (-1.14 - -0.46)	0.00	1042069 (932871,1186649)	39.56 (35.33,45)	-0.46 (-0.55 - -0.38)	0.00	107760 (74550,145649)	4.08 (2.83,5.56)	-0.48 (-0.57 - -0.39)	0.00
	Female	402536 (271112,603361)	35.22 (23.85,52.71)	-0.62 (-0.96 - -0.28)	0.00	417460 (369986,472946)	31.36 (27.73,35.66)	-0.39 (-0.50 - -0.28)	0.00	42709 (28734,58780)	3.21 (2.15,4.42)	-0.41 (-0.53 - -0.30)	0.00
	Male	711585 (534928,948319)	58.62 (44.13,78.56)	-0.83 (-1.12 - -0.54)	0.00	624608 (558123,723660)	47.76 (42.55,55.34)	-0.50 (-0.58 - -0.43)	0.00	65051 (45314,88059)	4.96 (3.45,6.71)	-0.52 (-0.59 - -0.44)	0.00
Andean Latin America	Both	39736 (29628,53947)	58.86 (43.83,80.15)	-1.33 (-1.52 - -1.14)	0.00	26551 (23574,30236)	41.2 (36.7,46.85)	-0.61 (-0.69 - -0.52)	0.00	2760 (1869,3833)	4.27 (2.9,5.95)	-0.62 (-0.72 - -0.52)	0.00
	Female	11515 (8219,16660)	35.42 (25.26,51.22)	-0.89 (-1.04 - -0.75)	0.00	8587 (7525,9831)	26.32 (23.1,30.07)	-0.46 (-0.52 - -0.39)	0.00	889 (586,1264)	2.72 (1.8,3.87)	-0.46 (-0.58 - -0.34)	0.00
	Male	28221 (20856,38196)	81.95 (60.77,110.65)	-1.54 (-1.76 - -1.32)	0.00	17964 (15877,20542)	56.75 (50.48,64.75)	-0.67 (-0.76 - -0.59)	0.00	1871 (1257,2629)	5.89 (3.99,8.22)	-0.69 (-0.80 - -0.58)	0.00
Australasia	Both	43740 (29112,66459)	169.16 (110.88,257.11)	-0.64 (-0.68 - -0.59)	0.00	72520 (65943,80854)	185.86 (168.13,210.1)	-0.67 (-0.70 - -0.65)	0.00	7462 (5028,10283)	19.32 (12.96,26.67)	-0.68 (-0.70 - -0.65)	0.00
	Female	15503 (10158,23585)	123.17 (79.86,190.89)	-0.16 (-0.22 - -0.11)	0.00	26924 (24465,30122)	133.41 (119.63,152.08)	-0.24 (-0.28 - -0.20)	0.00	2746 (1845,3805)	13.79 (9.27,19.17)	-0.23 (-0.27 - -0.18)	0.00
	Male	28237 (18730,42141)	214.08 (141.6,320.41)	-0.87 (-0.91 - -0.83)	0.00	45596 (41054,50648)	240.44 (214.97,269.51)	-0.92 (-0.94 - -0.90)	0.00	4716 (3151,6469)	25.08 (16.57,34.46)	-0.91 (-0.95 - -0.87)	0.00
Caribbean	Both	32821 (24669,44113)	71.8 (53.8,96.54)	0.25 (-0.14 - 0.65)	0.20	26750 (22911,33408)	52.84 (44.79,66.59)	0.61 (0.34 - 0.87)	0.00	2754 (1877,3865)	5.45 (3.69,7.7)	0.57 (0.37 - 0.77)	0.00
	Female	9936 (7185,13796)	44.28 (31.63,61.67)	0.31 (-0.21 - 0.84)	0.24	10989 (8442,16671)	41.95 (31.83,65.01)	1.22 (0.31 - 2.14)	0.01	1117 (709,1762)	4.28 (2.71,6.88)	1.21 (0.86 - 1.56)	0.00
	Male	22884 (17089,30710)	99.22 (73.92,133.74)	0.20 (-0.13 - 0.54)	0.22	15761 (13916,17776)	64.15 (56.32,72.75)	0.28 (0.09 - 0.46)	0.00	1637 (1100,2225)	6.66 (4.47,9.05)	0.19 (-0.00 - 0.38)	0.06
Central Asia	Both	78307 (57928,106414)	81.1 (60.06,110.33)	-1.02 (-1.32 - -0.73)	0.00	51255 (45992,57877)	54.79 (49.37,61.62)	-0.72 (-1.01 - -0.43)	0.00	5355 (3546,7364)	5.7 (3.8,7.84)	-0.73 (-0.93 - -0.53)	0.00
	Female	21343 (15327,31251)	45.16 (32.36,66.1)	-0.85 (-1.15 - -0.56)	0.00	16399 (14561,18598)	33.98 (30.17,38.54)	-0.66 (-0.84 - -0.48)	0.00	1700 (1123,2361)	3.51 (2.33,4.88)	-0.65 (-0.84 - -0.47)	0.00

	Male	56964 (42160,77196)	116.46 (86.31,157.75)	-1.13 (-1.43 - -0.83)	0.00	34855 (31058,39574)	78.06 (69.96,87.94)	-0.82 (-1.07 - -0.56)	0.00	3655 (2428,5046)	8.14 (5.45,11.18)	-0.82 (-1.06 - -0.58)	0.00
Central Europe	Both	137494 (95397,195478)	145.61 (101.23,208.27)	-1.10 (-1.20 - -1.00)	0.00	141608 (128995,157335)	90.48 (80.27,104.13)	-0.92 (-1.08 - -0.77)	0.00	14515 (9774,20064)	9.37 (6.15,13.08)	-0.91 (-1.07 - -0.75)	0.00
	Female	39338 (27148,59516)	88.15 (60.81,132.26)	-0.81 (-0.91 - -0.70)	0.00	51118 (46052,56948)	60.22 (52.99,69.19)	-0.78 (-0.93 - -0.62)	0.00	5188 (3448,7198)	6.22 (3.96,8.62)	-0.76 (-0.91 - -0.60)	0.00
	Male	98155 (68212,139400)	200.82 (138.54,287.84)	-1.23 (-1.33 - -1.13)	0.00	90491 (82124,101134)	122.89 (108.75,141.51)	-1.02 (-1.19 - -0.84)	0.00	9327 (6325,12751)	12.73 (8.44,17.8)	-1.01 (-1.18 - -0.84)	0.00
Central Latin America	Both	185817 (134240,257233)	75.01 (53.73,104.68)	-1.24 (-1.71 - -0.78)	0.00	139962 (125590,159057)	54.11 (48.37,61.49)	-1.23 (-1.38 - -1.09)	0.00	14514 (10019,19849)	5.6 (3.86,7.66)	-1.23 (-1.38 - -1.08)	0.00
	Female	59251 (39065,89593)	49.87 (32.84,75.14)	-0.98 (-1.49 - -0.48)	0.00	47161 (41374,54879)	35.31 (30.74,41.2)	-1.04 (-1.23 - -0.85)	0.00	4866 (3257,6817)	3.64 (2.42,5.1)	-1.04 (-1.22 - -0.85)	0.00
	Male	126566 (94506,171306)	101.29 (75.46,137.67)	-1.56 (-2.27 - -0.84)	0.00	92801 (83247,105681)	75.05 (67.46,85.31)	-1.26 (-1.41 - -1.11)	0.00	9648 (6664,13234)	7.78 (5.38,10.67)	-1.26 (-1.42 - -1.11)	0.00
Central Sub-Saharan Africa	Both	56603 (42905,74532)	35.88 (27.58,47.43)	-0.20 (-3.51 - 3.23)	0.91	31106 (24225,42693)	32.92 (26.24,45.2)	0.20 (-0.19 - 0.59)	0.31	3233 (2233,4745)	3.38 (2.33,4.93)	0.20 (-0.17 - 0.58)	0.29
	Female	20624 (14928,28495)	26.01 (19.12,35.63)	-0.23 (-2.62 - 2.22)	0.85	11020 (8990,14745)	23.33 (19.42,31.54)	-0.05 (-0.36 - 0.26)	0.75	1142 (751,1634)	2.38 (1.61,3.44)	-0.04 (-0.32 - 0.25)	0.80
	Male	35980 (27336,47815)	45.75 (35.39,60.24)	-0.13 (-3.95 - 3.85)	0.95	20086 (15150,28576)	43 (33.11,60.68)	0.31 (-0.13 - 0.74)	0.17	2091 (1392,3115)	4.43 (3.01,6.57)	0.31 (-0.12 - 0.74)	0.16
East Asia	Both	512517 (351164,734828)	38.18 (26.22,54.71)	-0.24 (-0.71 - 0.24)	0.33	632243 (568134,700276)	33.49 (29.77,37.35)	-0.26 (-0.46 - -0.05)	0.01	65573 (43443,90561)	3.48 (2.32,4.83)	-0.28 (-0.49 - -0.06)	0.01
	Female	198163 (122317,311725)	30.56 (18.86,48.65)	-0.35 (-1.15 - 0.45)	0.39	281848 (250964,315903)	29.35 (26,33.09)	-0.32 (-0.56 - -0.09)	0.01	28915 (18982,40090)	3.03 (2,4.21)	-0.35 (-0.61 - -0.09)	0.01
	Male	314354 (227535,438843)	45.1 (32.45,63.24)	-0.14 (-0.46 - 0.19)	0.42	350395 (314411,386817)	37.38 (33.29,41.6)	-0.20 (-0.39 - -0.00)	0.05	36658 (24513,50783)	3.91 (2.59,5.45)	-0.21 (-0.42 - -0.01)	0.04
Eastern Europe	Both	225307 (162194,317463)	124.29 (89.98,173.74)	-1.09 (-1.36 - -0.81)	0.00	240962 (219050,264675)	88.17 (78.83,99.32)	-0.91 (-0.98 - -0.84)	0.00	24741 (16477,33947)	9.11 (5.96,12.59)	-0.91 (-0.98 - -0.85)	0.00
	Female	64938 (44954,97020)	74.75 (51.51,111.66)	-0.71 (-0.90 - -0.53)	0.00	87678 (78737,97823)	56.89 (50.11,64.69)	-0.70 (-0.77 - -0.62)	0.00	8928 (5939,12354)	5.86 (3.78,8.19)	-0.70 (-0.77 - -0.63)	0.00
	Male	160368 (116741,222160)	174.51 (126.9,240.43)	-1.23 (-1.54 - -0.93)	0.00	153283 (139328,168364)	127.4 (114.42,143.11)	-1.05 (-1.12 - -0.99)	0.00	15812 (10548,21651)	13.15 (8.75,18.08)	-1.06 (-1.15 - -0.98)	0.00

Eastern Sub-Saharan Africa	Both	194153 (143423,270711)	39.49 (29.5,54.19)	-3.26 (-7.06 - 0.71)	0.11	104542 (79917,154032)	36.49 (28.07,54.48)	-0.67 (-1.20 - -0.13)	0.01	10878 (7356,16593)	3.75 (2.56,5.66)	-0.66 (-1.20 - -0.11)	0.02
	Female	70671 (51270,98155)	28.44 (20.77,39.54)	-1.58 (-2.12 - -1.05)	0.00	38218 (30015,55276)	26.21 (20.67,39.48)	-0.68 (-1.33 - -0.03)	0.04	3962 (2690,5807)	2.67 (1.8,4.05)	-0.46 (-0.79 - -0.13)	0.01
	Male	123482 (89917,175198)	50.9 (37.75,70.83)	-3.82 (-8.21 - 0.78)	0.10	66324 (49800,95323)	47.34 (35.54,68.88)	-0.66 (-1.12 - -0.20)	0.00	6917 (4608,10831)	4.89 (3.31,7.5)	-0.65 (-1.12 - -0.18)	0.01
High-income Asia Pacific	Both	106240 (75659,150514)	72.98 (51.39,104.27)	-1.20 (-1.43 - -0.97)	0.00	256099 (237161,279443)	88.43 (81.04,97.7)	-1.20 (-1.23 - -1.16)	0.00	26387 (17975,36057)	9.28 (6.21,12.83)	-1.19 (-1.23 - -1.16)	0.00
	Female	39624 (27441,57322)	56.16 (38.65,81.71)	-0.99 (-1.34 - -0.65)	0.00	104704 (96465,113933)	68.02 (61.89,75.25)	-0.99 (-1.03 - -0.94)	0.00	10695 (7250,14707)	7.11 (4.75,9.88)	-0.99 (-1.06 - -0.92)	0.00
	Male	66616 (47403,94110)	89.01 (62.44,125.87)	-1.31 (-1.44 - -1.18)	0.00	151395 (138672,166598)	109.39 (99.94,121.66)	-1.38 (-1.42 - -1.35)	0.00	15692 (10769,21426)	11.5 (7.79,15.85)	-1.38 (-1.42 - -1.33)	0.00
High-income North America	Both	211397 (155764,285658)	60.21 (44.37,82.21)	-1.31 (-1.56 - -1.06)	0.00	441108 (407523,473050)	85.29 (78.7,91.59)	-1.05 (-1.11 - -0.99)	0.00	44571 (30725,59437)	8.72 (5.97,11.72)	-1.10 (-1.17 - -1.03)	0.00
	Female	87210 (61870,123886)	47.62 (34.2,67.38)	-0.87 (-1.21 - -0.53)	0.00	196812 (177938,214709)	69.57 (63.47,76.01)	-0.62 (-0.73 - -0.50)	0.00	19677 (13469,26462)	7.06 (4.78,9.62)	-0.67 (-0.84 - -0.51)	0.00
	Male	124187 (93089,164749)	72.5 (54.05,95.7)	-1.56 (-1.70 - -1.43)	0.00	244296 (227164,262804)	101.61 (94.22,109.62)	-1.36 (-1.53 - -1.20)	0.00	24893 (17154,33492)	10.45 (7.13,14.02)	-1.39 (-1.55 - -1.23)	0.00
North Africa and Middle East	Both	522132 (380952,737439)	81.01 (59.04,114.4)	-0.11 (-1.07 - 0.86)	0.82	329689 (262141,446993)	56.43 (45.32,76.23)	-0.25 (-0.44 - -0.06)	0.01	34063 (23667,49232)	5.79 (4.03,8.32)	-0.27 (-0.46 - -0.09)	0.00
	Female	163976 (112157,242519)	53.18 (36.38,78.51)	-0.31 (-1.32 - 0.71)	0.55	105273 (86833,140673)	38.07 (31.72,50.59)	-0.26 (-0.39 - -0.13)	0.00	10777 (7228,15422)	3.87 (2.63,5.53)	-0.28 (-0.41 - -0.16)	0.00
	Male	358156 (257454,502288)	106.8 (76.79,149.88)	-0.03 (-1.05 - 1.01)	0.96	224416 (174537,307062)	73.48 (57.6,99.91)	-0.27 (-0.47 - -0.08)	0.01	23286 (16174,33554)	7.57 (5.26,10.95)	-0.30 (-0.49 - -0.10)	0.00
Oceania	Both	5772 (4176,8014)	38.84 (28.23,53.97)	-0.03 (-0.35 - 0.30)	0.87	3785 (3323,4342)	35.53 (31.82,39.95)	0.57 (0.43 - 0.71)	0.00	395 (266,549)	3.66 (2.51,5)	0.58 (0.53 - 0.63)	0.00
	Female	2848 (1761,4560)	39.72 (24.75,63.44)	0.29 (-0.15 - 0.74)	0.19	2003 (1678,2414)	39.43 (33.84,45.97)	0.87 (0.67 - 1.06)	0.00	208 (141,294)	4.04 (2.69,5.72)	0.88 (0.66 - 1.11)	0.00
	Male	2923 (2232,3719)	38.04 (29.23,48.46)	-0.34 (-0.63 - -0.05)	0.03	1782 (1587,2011)	31.66 (28.77,35.07)	0.23 (0.18 - 0.27)	0.00	186 (120,265)	3.28 (2.16,4.62)	0.22 (0.17 - 0.27)	0.00
South Asia	Both	752599 (523625,1048037)	39.87 (27.71,55.57)	-1.02 (-1.32 - -0.72)	0.00	597148 (527405,672529)	36.4 (32.33,40.95)	-0.44 (-0.52 - -0.36)	0.00	61301 (41809,84539)	3.7 (2.54,5.07)	-0.45 (-0.53 - -0.36)	0.00
	Female	341368 (218643,524052)	37.43 (23.99,57.25)	-1.02 (-1.20 - -0.84)	0.00	302292 (262046,348810)	37.53 (32.26,43.26)	-0.48 (-0.59 - -0.37)	0.00	30600 (20231,42442)	3.76 (2.48,5.17)	-0.49 (-0.59 - -0.39)	0.00

	Male	411231 (307135,541423)	42.12 (31.45,55.51)	-1.11 (-1.59 - -0.63)	0.00	294856 (260915,335838)	34.78 (31.03,39.08)	-0.45 (-0.54 - -0.35)	0.00	30700 (20607,41918)	3.59 (2.43,4.89)	-0.45 (-0.57 - -0.32)	0.00
Southeast Asia	Both	283453 (215917,379489)	40.93 (31.26,54.7)	-0.97 (-1.26 - -0.68)	0.00	235505 (205956,278311)	33.68 (29.53,39.74)	-0.71 (-0.99 - -0.42)	0.00	24462 (17010,33526)	3.48 (2.42,4.79)	-0.71 (-0.98 - -0.45)	0.00
	Female	101740 (73589,142172)	30.32 (22.13,42.22)	-0.89 (-1.23 - -0.55)	0.00	93322 (80363,114827)	26.49 (22.81,32.53)	-0.61 (-0.76 - -0.46)	0.00	9622 (6556,13380)	2.72 (1.84,3.79)	-0.63 (-0.79 - -0.46)	0.00
	Male	181713 (141330,237832)	51.2 (39.75,66.96)	-1.05 (-1.31 - -0.80)	0.00	142183 (123799,170721)	41.07 (36.03,48.71)	-0.84 (-1.09 - -0.59)	0.00	14840 (10226,20665)	4.26 (2.94,5.89)	-0.84 (-1.09 - -0.59)	0.00
Southern Latin America	Both	83454 (55651,124922)	131.18 (87.7,195.96)	-0.30 (-0.39 - -0.21)	0.00	120864 (110017,133589)	156.81 (142.16,173.66)	-0.40 (-0.44 - -0.37)	0.00	12594 (8487,17206)	16.39 (10.97,22.43)	-0.41 (-0.45 - -0.36)	0.00
	Female	23446 (15571,36041)	76.91 (50.76,117.43)	0.03 (-0.07 - 0.13)	0.56	36442 (33424,40162)	90.02 (81.95,99.9)	-0.11 (-0.13 - -0.08)	0.00	3757 (2577,5270)	9.34 (6.35,13.14)	-0.10 (-0.16 - -0.04)	0.00
	Male	60007 (40051,89631)	185.56 (123.35,277.53)	-0.42 (-0.48 - -0.37)	0.00	84422 (75751,93678)	230.67 (207.34,256.31)	-0.54 (-0.58 - -0.51)	0.00	8837 (5923,12149)	24.16 (16.17,33.19)	-0.55 (-0.58 - -0.51)	0.00
Southern Sub-Saharan Africa	Both	31260 (24154,40825)	37.1 (28.6,48.49)	-1.08 (-1.21 - -0.94)	0.00	24615 (22263,27426)	33.9 (30.83,37.48)	-1.23 (-1.31 - -1.15)	0.00	2550 (1763,3495)	3.49 (2.42,4.76)	-1.26 (-1.33 - -1.19)	0.00
	Female	9313 (6888,12595)	22.22 (16.44,29.97)	-1.34 (-1.51 - -1.17)	0.00	7531 (6841,8362)	19.69 (17.93,21.75)	-1.54 (-1.62 - -1.46)	0.00	775 (536,1067)	2.01 (1.4,2.75)	-1.56 (-1.63 - -1.49)	0.00
	Male	21947 (16780,28461)	52.45 (40.13,68.2)	-1.01 (-1.16 - -0.86)	0.00	17084 (15377,19213)	50.92 (46.21,56.96)	-1.09 (-1.16 - -1.02)	0.00	1776 (1220,2471)	5.24 (3.67,7.2)	-1.11 (-1.21 - -1.02)	0.00
Tropical Latin America	Both	154468 (113090,212375)	70.24 (51.11,97.36)	-0.91 (-1.13 - -0.68)	0.00	132533 (118016,148462)	53.1 (46.99,59.85)	-0.79 (-0.85 - -0.73)	0.00	13656 (9390,18757)	5.47 (3.77,7.53)	-0.79 (-0.85 - -0.73)	0.00
	Female	44031 (28499,67915)	40.74 (26.36,63.09)	-0.98 (-1.18 - -0.79)	0.00	42904 (37182,49192)	32.61 (28.14,37.52)	-0.88 (-0.95 - -0.82)	0.00	4365 (2857,6033)	3.33 (2.16,4.64)	-0.89 (-0.97 - -0.81)	0.00
	Male	110437 (82297,148280)	99.93 (74.2,134.31)	-0.88 (-1.23 - -0.52)	0.00	89629 (80135,101154)	75.35 (67.19,85.17)	-0.74 (-0.81 - -0.66)	0.00	9291 (6467,12769)	7.79 (5.41,10.71)	-0.73 (-0.81 - -0.65)	0.00
Western Europe	Both	278486 (196994,406937)	78.75 (54.61,115.31)	-0.74 (-0.81 - -0.67)	0.00	573343 (526653,624885)	91.75 (83.49,101.92)	-0.84 (-0.87 - -0.81)	0.00	58853 (40413,79629)	9.57 (6.48,13.16)	-0.84 (-0.87 - -0.81)	0.00
	Female	103525 (66681,161649)	58.07 (37.53,91.96)	-0.26 (-0.30 - -0.22)	0.00	235228 (214178,257930)	68.84 (62.01,76.38)	-0.42 (-0.44 - -0.39)	0.00	23789 (16260,32783)	7.11 (4.8,9.86)	-0.42 (-0.47 - -0.38)	0.00
	Male	174961 (126433,246910)	98.63 (70.76,140.56)	-0.97 (-1.04 - -0.90)	0.00	338114 (310933,369614)	115.23 (105.01,127.71)	-1.11 (-1.15 - -1.07)	0.00	35063 (24023,47915)	12.08 (8.17,16.58)	-1.11 (-1.15 - -1.07)	0.00

Western Sub-Saharan Africa	Both	198946 (153358,263367)	34.85 (26.98,46.29)	-0.22 (-0.83 - 0.39)	0.48	91859 (79223,107694)	27.22 (24.42,30.91)	-0.14 (-0.27 - -0.02)	0.03	9633 (6574,13247)	2.82 (1.99,3.77)	-0.13 (-0.24 - -0.02)	0.02
	Female	76847 (56322,104355)	26.09 (19.36,35.45)	-0.50 (-0.76 - -0.24)	0.00	36234 (31202,42313)	20.95 (18.7,23.68)	-0.26 (-0.38 - -0.14)	0.00	3772 (2506,5367)	2.14 (1.5,2.92)	-0.26 (-0.38 - -0.14)	0.00
	Male	122099 (95251,157878)	44.26 (34.79,56.83)	-0.08 (-0.79 - 0.64)	0.83	55625 (47687,65696)	34.14 (30.64,39.11)	-0.01 (-0.08 - 0.06)	0.79	5861 (4016,8097)	3.56 (2.53,4.78)	0.01 (-0.06 - 0.07)	0.84

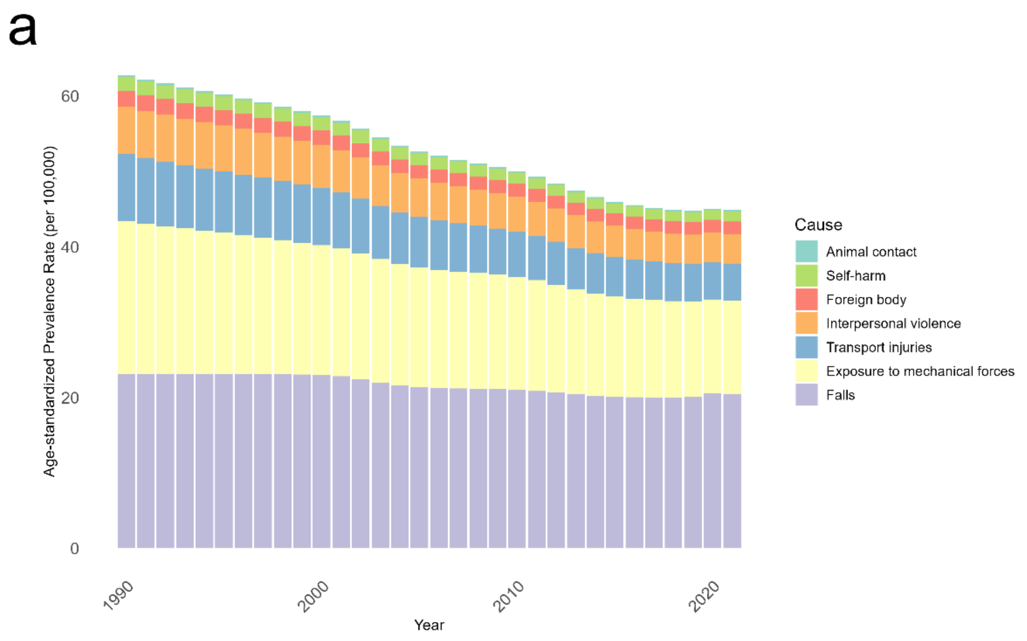

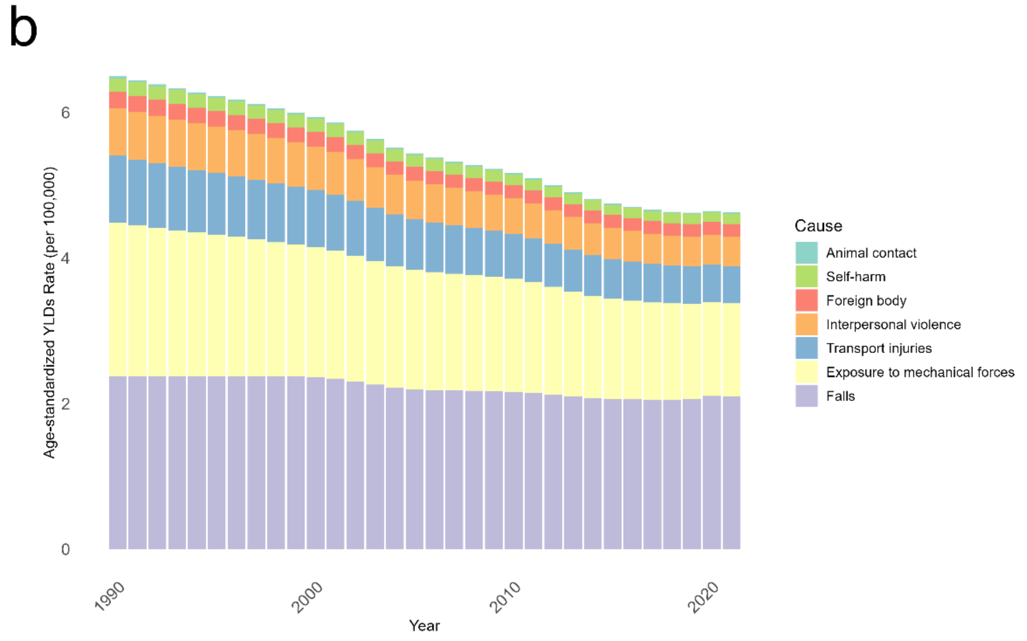

Figure 4 extension.

a: Cause composition of age-standardized prevalence of nerve injury by GBD for both genders from 1990 to 2021.b: Cause composition of age-standardized YLDs of nerve injury by GBD for both genders from 1990 to 2021.

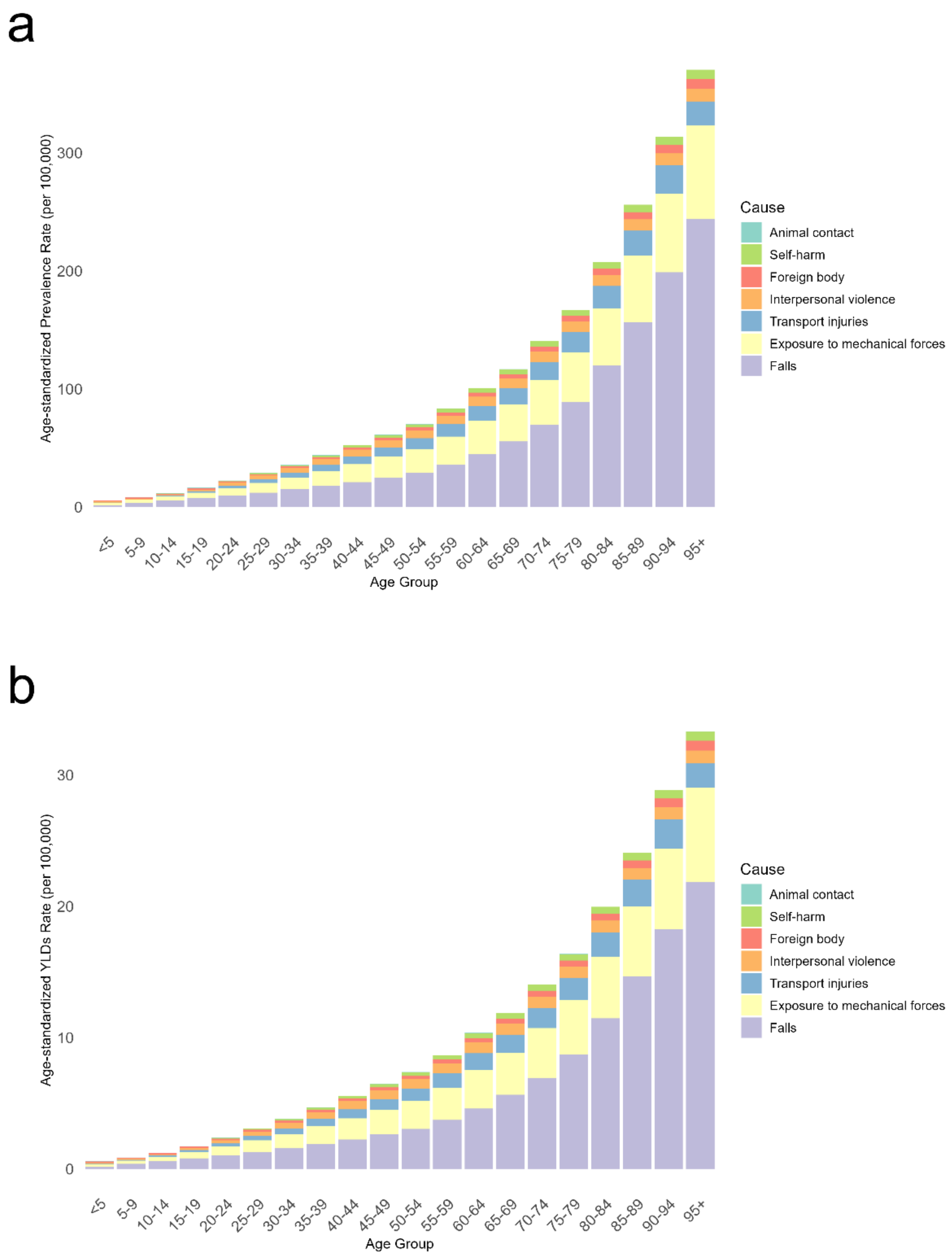

Figure 5 extension.

a: Cause composition of age - standardized prevalence of nerve injury for both genders in different age groups in 2021.b: Cause composition of age - standardized YLDs of nerve injury for both genders in different age groups in 2021.

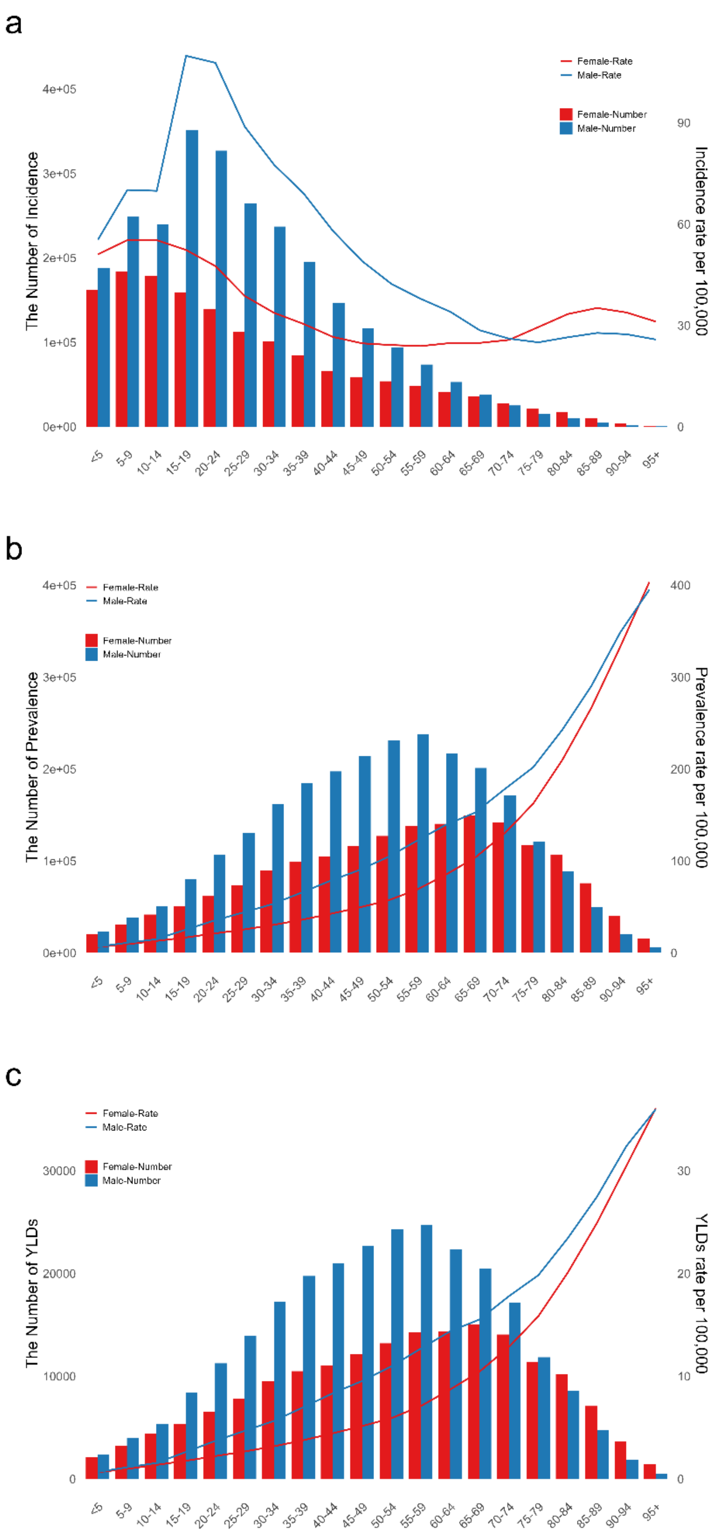

Fig.S1 The numbers and rates of nerve injury in different age groups by gender. a: The incidence numbers and rates of nerve injury in different age groups by gender. b: The prevalence numbers and rates of nerve injury in different age groups by gender. c: The YLDs numbers and rates of nerve injury in different age groups by gender.

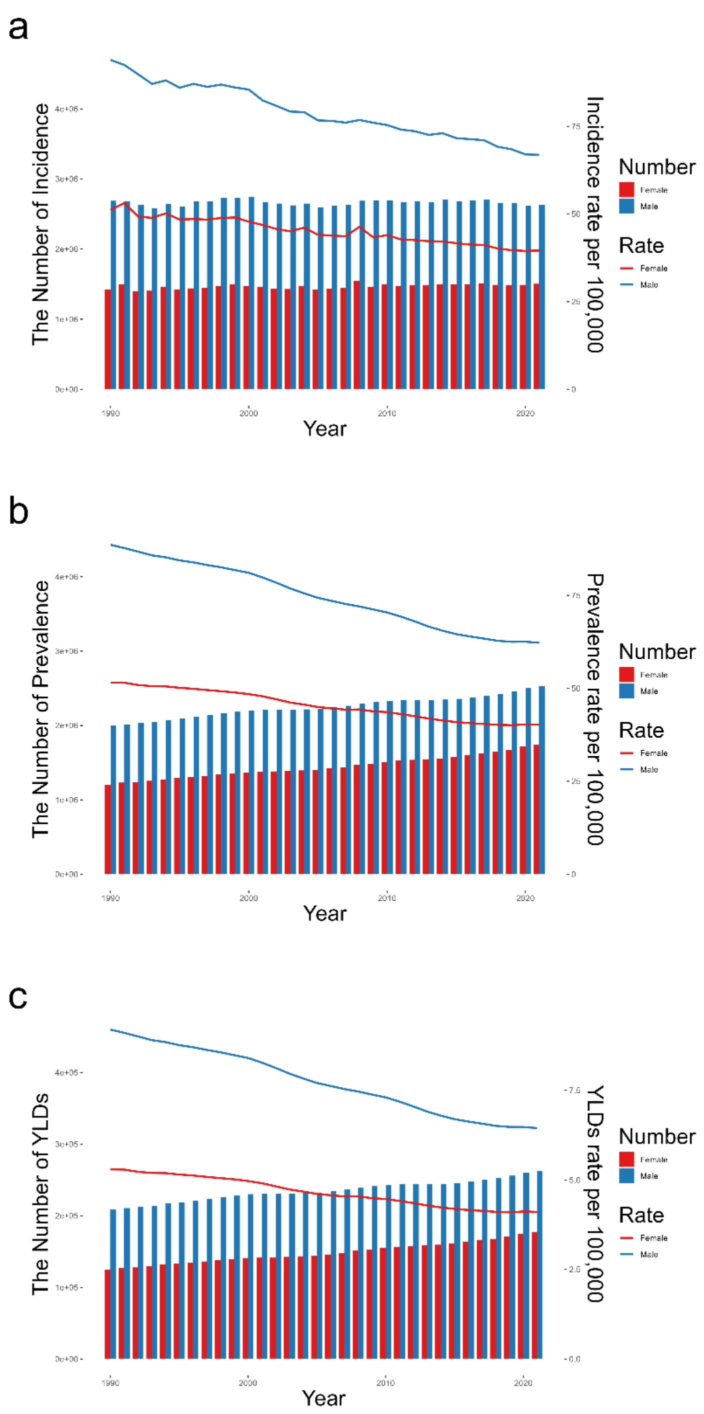

Fig.S2 The numbers and rates of nerve injury from 1990 to 2021 by gender. a: The incidence numbers and rates of nerve injury from 1990 to 2021 by gender. b: The prevalence numbers and rates of nerve injury from 1990 to 2021 by gender. c: The YLDs numbers and rates of nerve injury from 1990 to 2021 by gender.

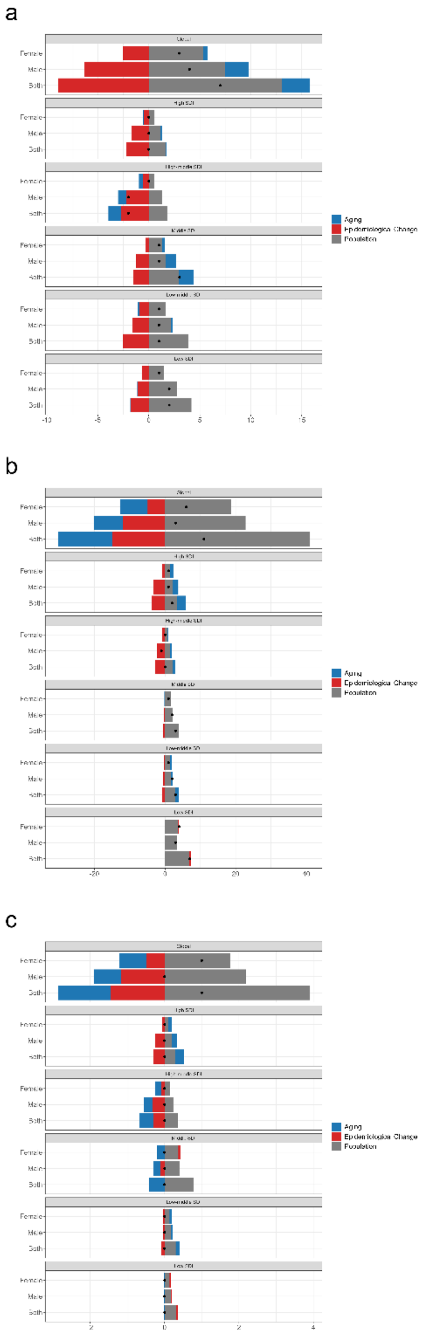

Fig.S3 Decomposition analysis of the impacts of aging, epidemiological changes and population on females, males and both genders in global and different SDI regions. a: Decomposition analysis of the impacts of aging, epidemiological changes and population on the incidence of females, males and both genders in global and different SDI regions. b: Decomposition analysis of the impacts of aging, epidemiological changes and population on the prevalence of females, males and both genders in global and different SDI regions. c: Decomposition analysis of the impacts of aging, epidemiological changes and population on the YLDs of females, males and both genders in global and different SDI regions

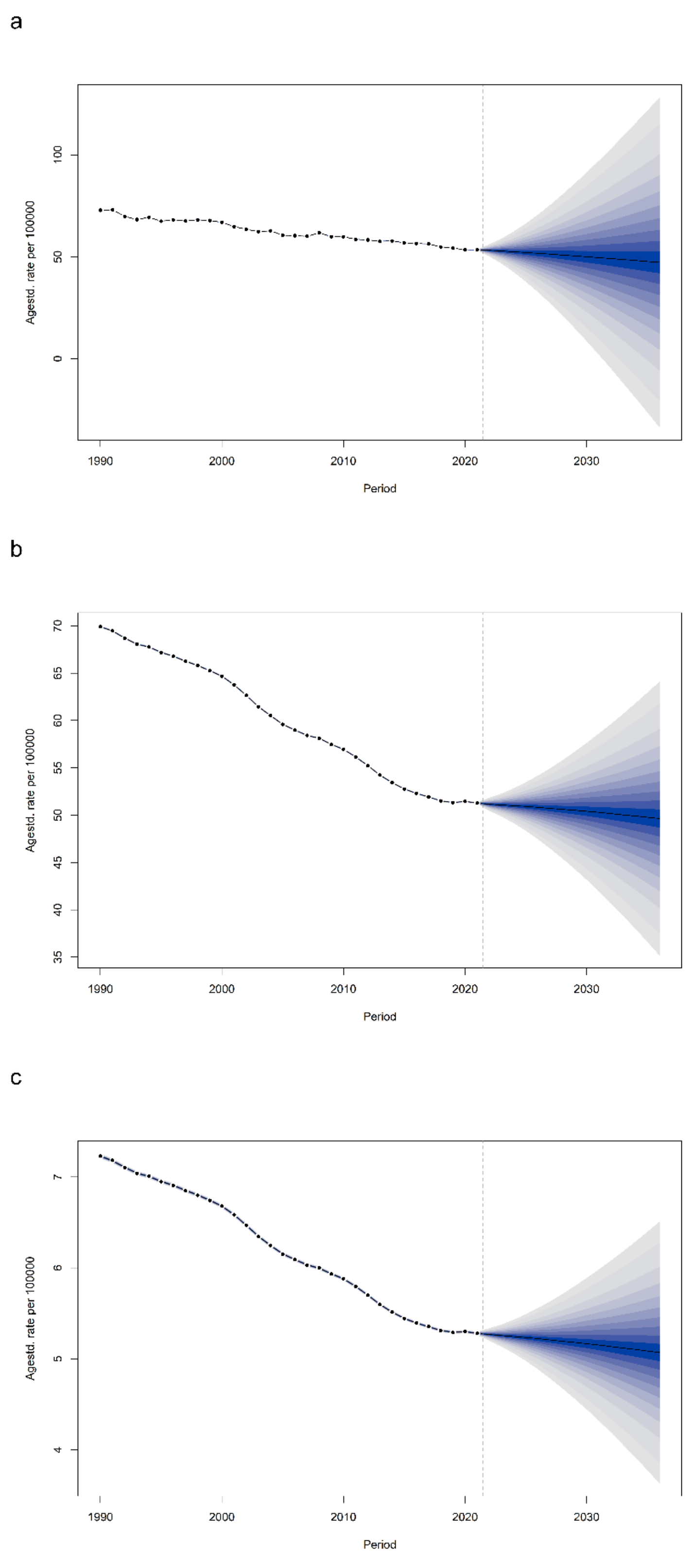

Fig.S4 Trend and prediction of age-standardized rate of nerve injury from 1990 to 2030. a: Trend and prediction of incidence age-standardized rate of nerve injury from 1990 to 2030. b: Trend and prediction of prevalence age-standardized rate of nerve injury from 1990 to 2030. c: Trend and prediction of YLDs age-standardized rate of nerve injury from 1990 to 2030.

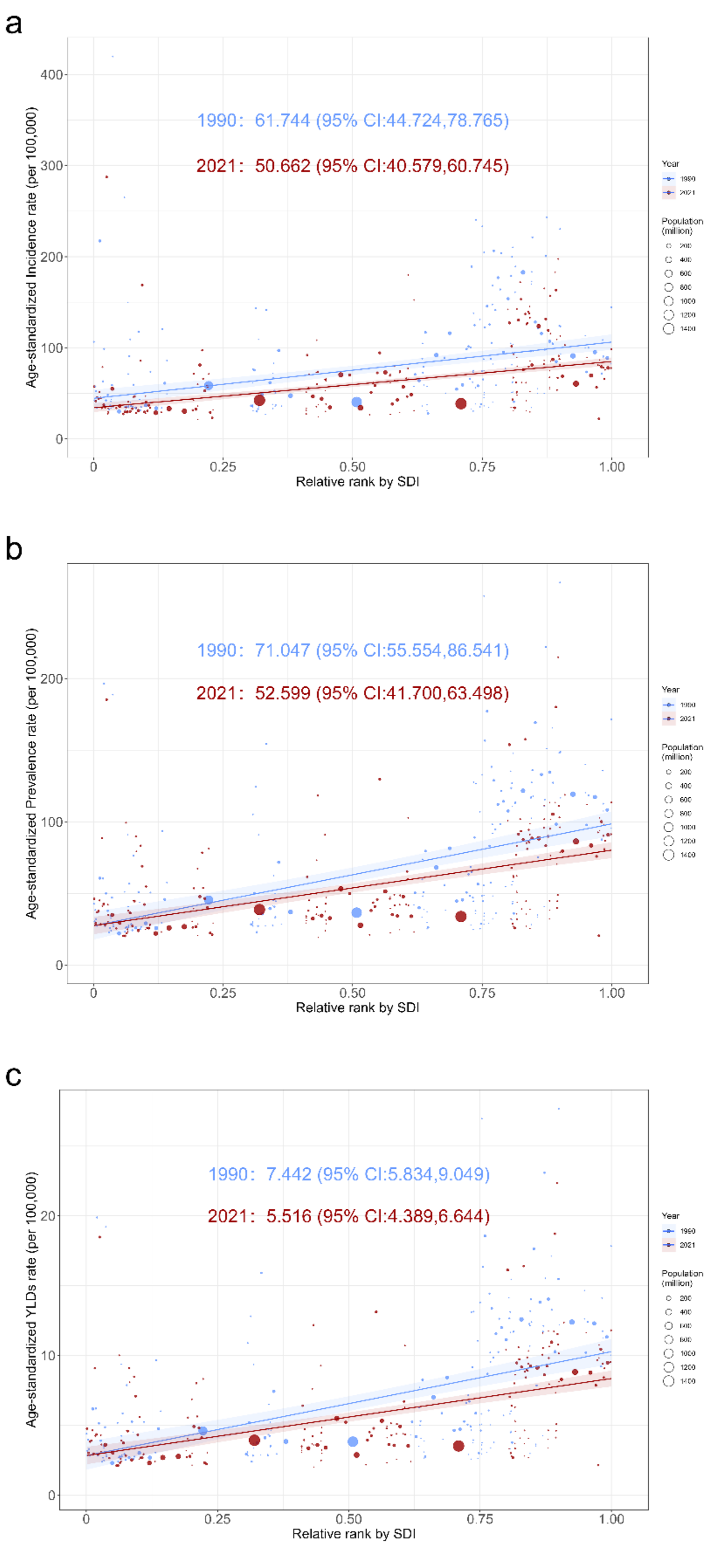

Fig.S5 Comparison of age-standardized rate of nerve injury by SDI relative rank between 1990 and 2021. a: Comparison of age-standardized incidence rate of nerve injury by SDI relative rank between 1990 and 2021. b: Comparison of age-standardized prevalence rate of nerve injury by SDI relative rank between 1990 and 2021. c: Comparison of age-standardized YLDs rate of nerve injury by SDI relative rank between 1990 and 2021.

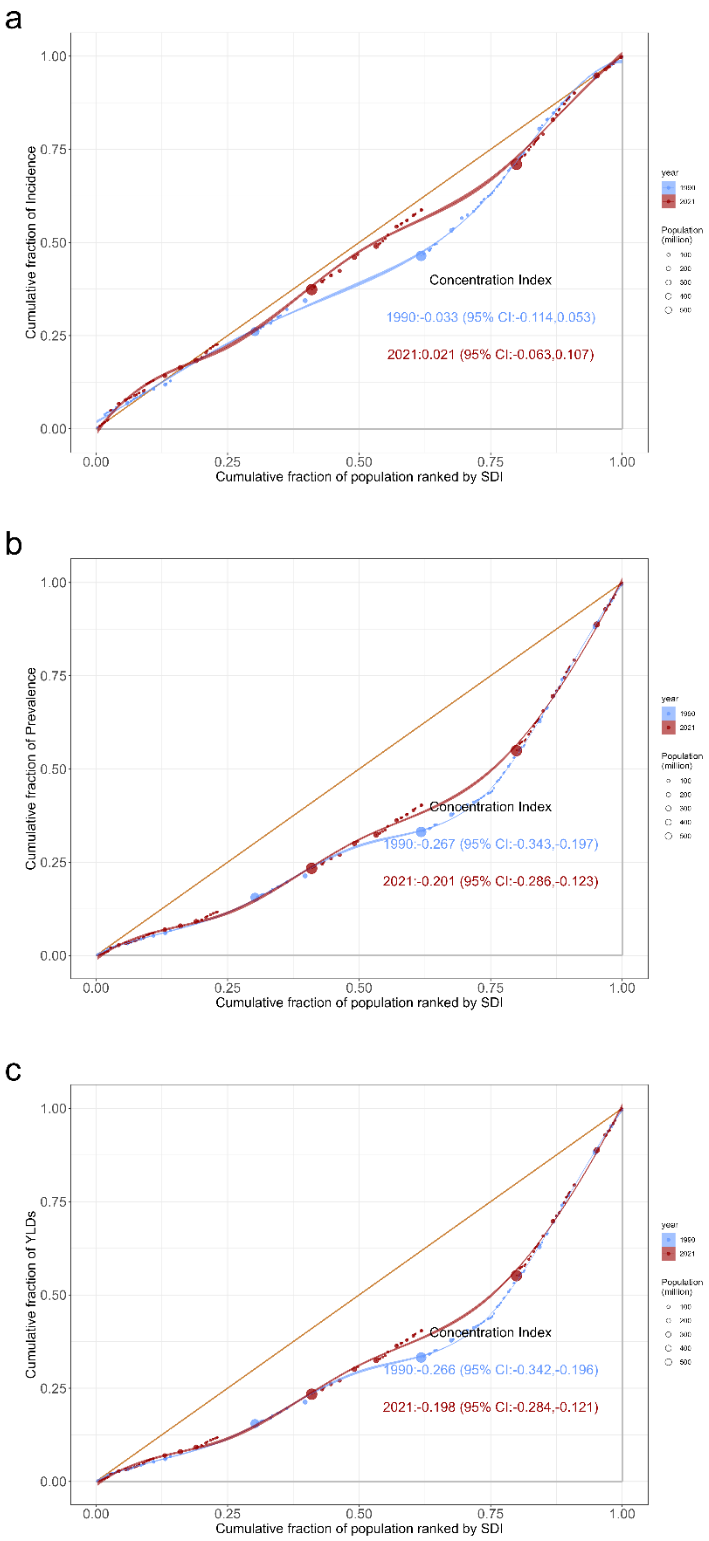

Fig.S6 Comparison cumulative fraction of nerve injury by SDI relative rank between 1990 and 2021. a: Comparison of the cumulative fraction of incidence of nerve injury by SDI relative rank between 1990 and 2021. b: Comparison of the cumulative fraction of prevalence of nerve injury by SDI relative rank between 1990 and 2021. Cc: Comparison of the cumulative fraction of YLDs of nerve injury by SDI relative rank between 1990 and 2021.

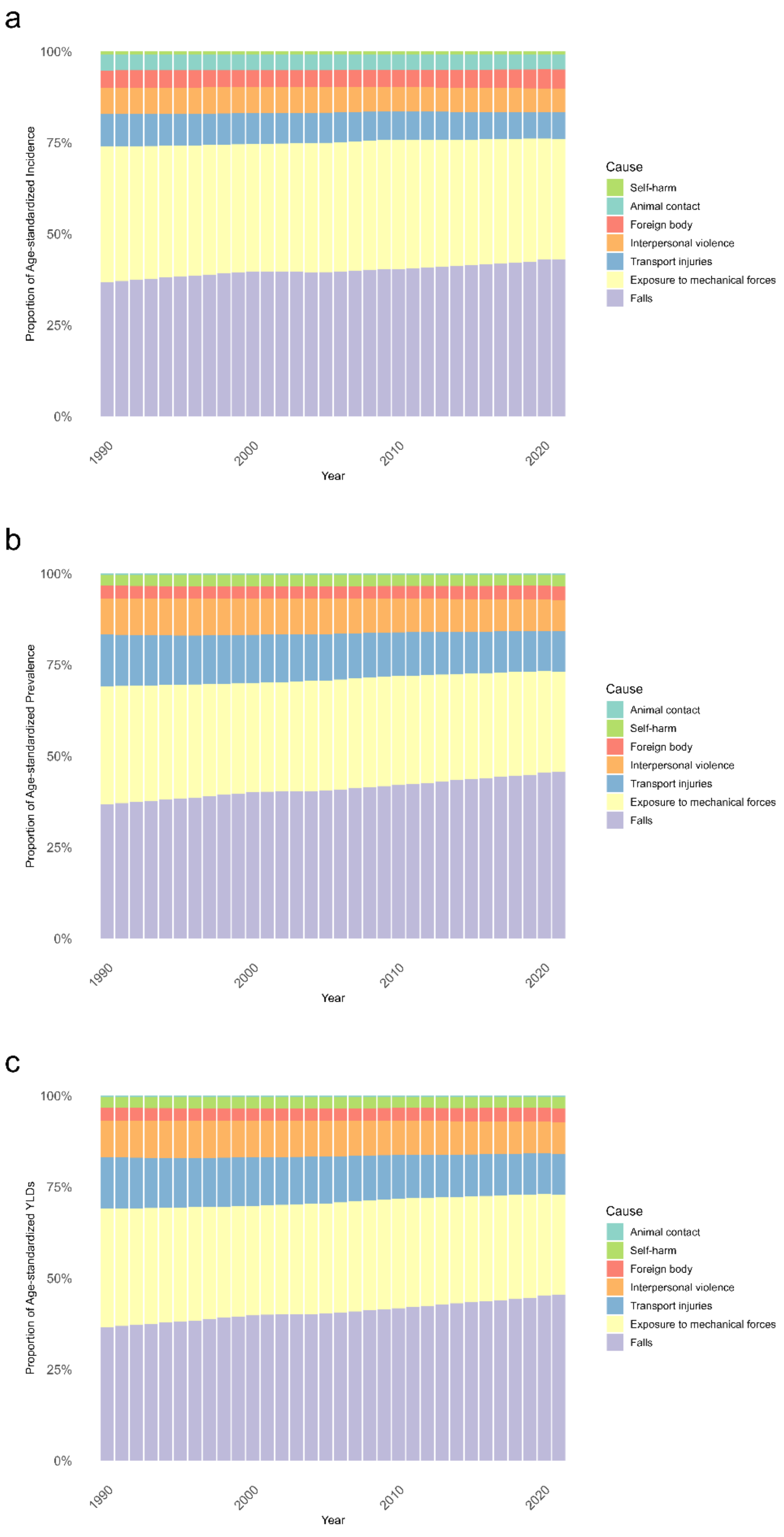

Fig.S7 Trend chart of age-standardized constituent ratios of nerve injuries caused by different etiologies from 1990 to 2020. a: Age-standardized constituent ratio of nerve injury incidence by etiology from 1990 to 2020. b: Age-standardized constituent ratio of nerve injury prevalence by etiology from 1990 to 2020.c: Age-standardized constituent ratio of nerve injury YLDs by etiology from 1990 to 2020.
